# Hypoxia-Ischemia Induced Age-Dependent Gene Transcription Effects at Two Development Stages in the Neonate Mouse Brain

**DOI:** 10.3389/fnmol.2020.587815

**Published:** 2020-12-02

**Authors:** Nicolas Dupré, Céline Derambure, Bérénice Le Dieu-Lugon, Michelle Hauchecorne, Yannick Detroussel, Bruno J. Gonzalez, Stéphane Marret, Philippe Leroux

**Affiliations:** ^1^INSERM-UMR 1245, Team 4, Epigenetics and Physiopathology of Neurodevelopmental Brain Lesions, Faculté de Médecine et de Pharmacie, Normandie Université, Rouen, France; ^2^INSERM-UMR 1245, Team 1, Genetic Predisposition to Cancer, Faculté de Médecine et de Pharmacie, Normandie Université, Rouen, France; ^3^CURIB, Faculté des Sciences et Techniques, Normandie Université, Mont-Saint-Aignan, France; ^4^Neonatal Pediatrics, Intensive Care Unit and Neuropediatrics, Rouen University Hospital, Rouen, France

**Keywords:** brain-development, cholesterol-biosynthesis, encephalopathy, hypoxia-ischemia, inflammation, mouse, neonate, transcriptome

## Abstract

Human brain lesions in the perinatal period result in life-long neuro-disabilities impairing sensory-motor, cognitive, and behavior functions for years. Topographical aspects of brain lesions depend on gestational age at the time of insult in preterm or term infants and impaired subsequent steps of brain development and maturation. In mice, the Rice-Vannucci procedure of neonate hypoxia-ischemia (HI) was used at 5 days (P5) or P10, mimicking the development of 30 week-gestation fetus/preterm newborn, or full-term infant, respectively. Transcription response to HI was assessed at 3, 6, 12, and 24 h after insult, using micro-array technology. Statistical Pathway and Gene Ontology terms enrichments were investigated using DAVID^®^, Revigo^®^ and Ingenuity Pathway Analysis (IPA^®^) to identify a core of transcription response to HI, age-specific regulations, and interactions with spontaneous development. Investigations were based on direction, amplitude, and duration of responses, basal expression, and annotation. Five major points deserve attention; (i) inductions exceeded repressions (60/40%) at both ages, (ii) only 20.3% (393/1938 records) were common to P5 and P10 mice, (iii) at P5, HI effects occurred early and decreased 24 h after insult whereas they were delayed at P10 and increased 24 h after insult, (iv) common responses at P5 and P10 involved inflammation, immunity, apoptosis, and angiogenesis. (v) age-specific effects occurred with higher statistical significance at P5 than at P10. Transient repression of 12 genes encoding cholesterol biosynthesis enzymes was transiently observed 12 h after HI at P5. Synaptogenesis appeared inhibited at P5 while induced at P10, showing reciprocal effects on glutamate receptors. Specific involvement of Il-1 (interleukin-1) implicated in the firing of inflammation was observed at P10. This study pointed out age-differences in HI responses kinetics, e.g., a long-lasting inflammatory response at P10 compared to P5. Whether the specific strong depression of cholesterol biosynthesis genes that could account for white matter-specific vulnerability at P5 or prevent delayed inflammation needs further investigation. Determination of putative involvement of Il-1 and the identification of upstream regulators involved in the delayed inflammation firing at P10 appears promising routes of research in the understandings of age-dependent vulnerabilities in the neonatal brain.

## Introduction

Cerebral palsy is the most severe motor developmental disability in children, accompanied by sensorial deficits, cognitive, language, and behavioral impairments diversely associated. It is a complex disease due to entangled antenatal, perinatal, and postnatal noxious events. The topographical aspects of brain lesions visible with medical imagery, or in post-mortem tissues, show a clear relationship to post-conception (PC) age of infants (Volpe, 2009; Korzeniewski et al., 2018). The relationship between brain lesions and delayed children’s disabilities is less stringent, indicating that common causes have distinct brain consequences depending on brain maturity. Cerebellum and periventricular germinative area are prone to hemorrhage in extreme preterm neonates (before 28 PC weeks). White matter in preterms born at 28–34 PC weeks has a special vulnerability, clearly associated with oligodendroglia and connectome differentiation stage at this time, whereas gray matter appeared more sensitive in the late gestation and later in the postnatal period when *N*-methyl-*D*-aspartate (NMDA)-type glutamate receptors are functional in neurons. The common triggers recorded are ischemia when blood flow is decreased, oxidative stress and inflammation (Hagberg et al., 2015). Perfusion arrest and reperfusion are common in preterm having fluctuant hemodynamics and immature regulation of cerebral blow flow (Tuor and Grewal, 1994). Reperfusion leads to oxidative stress and inflammation.

Whether age specificities in the development of brain lesions and subsequent disabilities were due to interaction with the genetic development program, interference with maturation progress of particular cell lineage and/or induction of outlandish responses appears an exciting challenge. Animal models move toward a description of pertinent procedures that mimic tissue lesions and/or functional impairments. Less than the trigger used to provoke brain insults, the accuracy of the model is verified when insults recapitulate physiopathology at the tissue level, behavior, and chronology of effects. The use of 5-day old (P5) and P10 mice to induce brain lesions mimicking those observed in preterm and term infants is currently admitted (Johnston, 2005; Hagberg et al., 2015). The HI model proposed by Rice et al. (1981) in P7 rats, successfully transferred in P5 and P10 mice actually induced differential white/gray matter MRI signatures, microglial responses and delayed behavior impairments (Daher et al., 2018; Dupré et al., 2020). The brain continues to grow and maturate after birth beyond P10 in mice; neurons establish synaptic contacts, glia proliferate and parenchyma growth promotes angiogenesis. In parallel, intrinsic brain immune cells and developing immune system may affect the brain development trajectory (Bilbo and Schwarz, 2012). Brain tissue therefore, exhibit a clearly different background condition at the time of insult, underlying age-specific structural vulnerabilities and defense means, defining age-dependent substrata (Jiang and Nardelli, 2016; Adle-Biassette et al., 2017).

Very different environmental inputs affect the brain development program via either metabolic or sensorial modalities (Bolton and Bilbo, 2014; Vinall et al., 2014; Loewy and Jaschke, 2020). Direct epigenetic marks were reported on microglia after ischemic trauma (Fleiss and Gressens, 2012). Life-long effects of perinatal stress affect the microglial fate and secondarily neuronal and non-neuronal development (Paolicelli and Ferretti, 2017; Catale et al., 2020). Experimental HI in rodents appears a valuable approach integrating several noxious factors; surgical stress, oxygen deprivation, and rapid restoration, or thermal regulation, mimicking several contingent factors affecting human preterm and neonates (Rumajogee et al., 2016). Experimental HI in neonatal mice actually provokes the common brain noxious phenomenon of inflammation, exhibits age-dependent region vulnerability, especially on vessels and white matter, and leads to age-dependent disabilities (Leroux et al., 2014; Hagberg et al., 2015; Dupré et al., 2020). In front of the huge complexity of cell types and interactions, extracellular environment, development changes, it appears utopian to reach an understanding of the differences due to age in the effects of HI in the neonatal period using hypothesis-driven perspectives. Unbiased holistic omics approaches might be more pertinent toward this goal. As we showed previously, microvessel undergoes rapid strengthening, changes in receptors, transporters, and metabolism in the P5–P10 neonatal period in mice (Porte et al., 2017a,b). The present study was aimed at identifying the development of specific differences in response to HI in neonatal mice. The ultimate goal was to identify targets for putative age-adjusted intervention tools, in particular with therapeutic hypothermia which is now widely recommended by obstetrical and neonatal learned societies in case of brain asphyxia at birth in term newborn (Jacobs et al., 2013).

We performed transcriptome comparisons using whole-genome microarrays of HI versus naive mRNA in P5 and P10 mice brains. In parallel we followed spontaneous mRNA basal expression from P2 to P15 in naive animals, in order to (i) described differences in background gene expression depending on age, (ii) to characterize common, and age-dependent processes activated (repressed), at four-time intervals after the insult (from 3 to 24 h), (iii) to extract interferences of age with HI responses. Biostatistical analyses of enriched pathways and Gene ontology (GO) terms were undertaken to extract pertinent information. Inflammatory protein levels were assessed in parallel to gene expression in an attempt to establish technical validation and functional correlates.

## Materials and Methods

### Ethics Statement

NMRI mice (Naval Medical Research Institute) purchased from Janvier (Le Genest Saint Isle, France) were housed in controlled temperature, humidity, and day/night 12/12 h cycle, with water and food *ad libitum*. All procedures used were approved by the French “Ministère de l’Education Nationale de l’Enseignement Supérieur et de la Recherche” (Agreement 016800.02 on 10/13/2014) in accordance with French law (R.214-87 and R.214-126), INSERM recommendations in respect with the EU directive 2010/63/EU for animal experiments.

### Hypoxia-Ischemia Procedure

**FIGURE 1:**
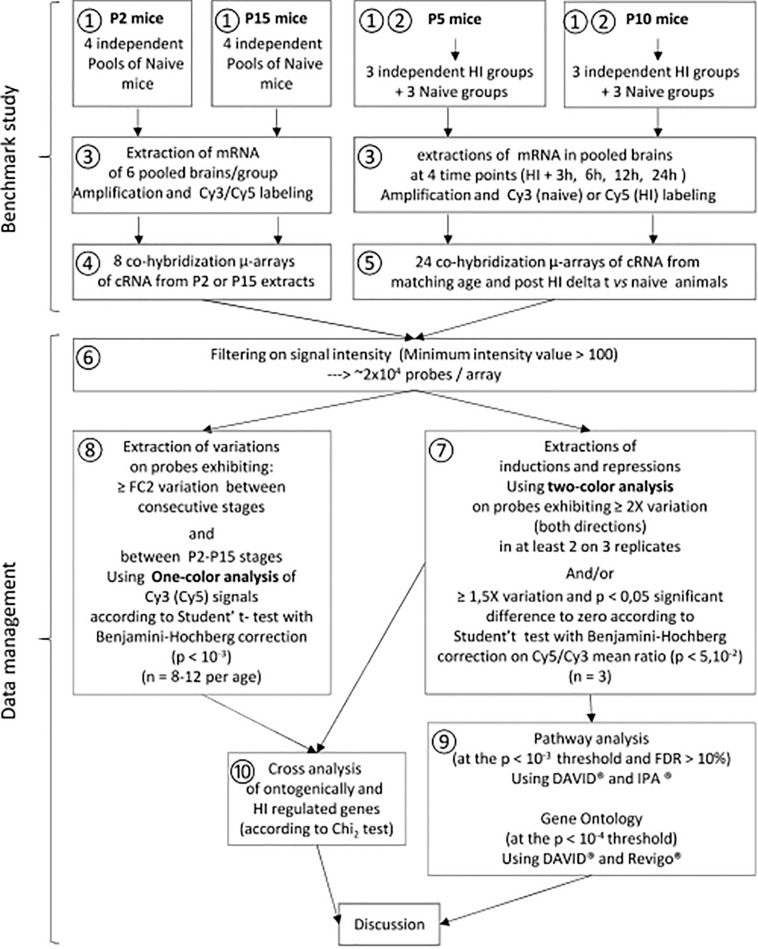
Transcriptome study experimental schedule. Spontaneous development **①** and effects of HI **②** were studied in parallel. Cy3 signals were used for development study at P5 and P10. **③** mRNA extraction amplification and labeling. **④** Co-hybridization of Cy3 labeled P2 and Cy5-P15 samples. **⑤** Co-hybridization of Cy3-naïve and Cy5-HI samples at P5 or P10. **⑥** Signal filtering on signal quality and basal expression at the same thresholds. **⑦** Extraction of HI effects at P5 or P10 using a GeneSpring^®^ two-color procedure. **⑧** Extraction of ontogenetic variations between P2, P5, P10, and P15 using a GeneSpring^®^ one-color procedure. **⑨** Biostatistical analyses of HI effects at P5 or P10 utilizing DAVID^®^, Revigo^®^, and IPA^®^. **⑩** Identification of convergences between spontaneous development and effects of HI at P5 or P10 using Excel and GraphPad. cRNA: Cy3 or Cy5 labeled complementary RNA from retro-transcribed DNA obtained from extracted mRNA.

The HI procedure derives from the procedure described in P7 rats (Rice et al., 1981) adapted to P5 or P10 mice (Dupré et al., 2020). Briefly, neonate mice were submitted to the right common carotid ligature under isoflurane anesthesia, followed by a 40 min exposure to restricted O2 (8%) (① in Figure 1). All the procedure was done under controlled temperature (36°C). Naive animals were used as control. A total of 336 animals were used for microarray study (① and ② in Figure 1) and 36 animals for Western blot experimentation and protein arrays (details in Supplementary Material).

### RNA Samples Preparation

Total RNA were extracted from brain hemispheres, ipsilateral to ligature at 3, 6, 12, or 24 h after HI procedure at P5 or P10 in sex-matched groups and from the right brain hemispheres of age- and time-matched naive animals (details in Supplementary Material). RNA samples at P2 and P15 required for the developmental study were collected in 8 separate pools each of 6 sex-matched animals at both stages (③ in Figure 1). Brain tissues were defrosted in 350 μl lysis buffer from NucleoSpin RNA Plus extraction kit (Macherey-Nagel, Hoerdt, France) and homogeneized using ceramic beads (1.4 mm Ozyme, Montigny le Bretonneaux) in a tissue lyser^®^ (Qiagen, Courtaboeuf, France) for 20 s at 50 hz. Total RNAs extraction was performed using Nucleospin RNA plus kit^®^ according to manufacturer instructions (Macherey-Nagel) in 350 μL and frozen at −80°C. The integrity and quantity of isolated mRNAs were assessed using the 2100 Bio-analyzer (Agilent Technologies, Santa Clara, CA, United States) and the Nanodrop device (Thermo Scientific, Wilmington, United States). See details in Supplementary Material. Pooled samples for microarray study were made with 5 μg of total RNA extracted from 6 ipsilateral hemispheres of sex matched injured or control mice at both ages and all time points.

### Transcriptome Analyses

Hundred nanogram of RNAs were labeled with cyanine-5 CTP (ischemic) or cyanine-3 CTP (control). After hybridization reaction using hybridization kit (Whole Mouse Genome Oligo 4 × 44K Microarray (G4845A, Agilent Technologies, Les Ulis, France). Co-hybridization was performed at 65°C for 17 h. After wash steps, the microarrays were scanned with a 5 μM pixel size using the DNA Microarray Scanner G2565CA (Agilent Technologies). Image analysis and extraction of raw and normalized signal intensities (Lowess) were performed with Feature Extraction Software 10.5.1.1 (Agilent Technologies). Data were analyzed with GeneSpring GX V.12.6 (Agilent Technologies).

Two-color comparative hybridization was performed to compare gene expression profiling at 4-time points after HI in injured hemispheres from P5 or P10 mice, with age-and time-matched naive animals (⑤ in Figure 1, details in Supplementary Material). Raw hybridization data, evaluated on every 5 μm array probe, was extracted and normalized (Lowess method), then transferred to Genespring^®^ for data processing and data mining (⑥ in Figure 1). Transcriptomic effects of HI were recorded on probes exhibiting a Cy3/Cy5 fold change (FC) > 2 in at least two of three replicates and probes exhibiting Cy3/Cy5 FC > 1.5 and p-value < 0.05 using Benjamini–Hochberg *t*-test against zero. Probes with hybridization level < 100 in the two fluorochromes were excluded (⑥ in Figure 1). Genes identified with several probes were examined using signals with the highest amplitude.

Basal expression along development process was assessed at P5 and P10 from Cy3 signals of controls in all array, and from mRNA extracted from naive mice at P2 and P15 (*n* = 8 independent pools of 6 brain hemispheres from separate litters). One-color analysis (Using Genespring^®^) in the complete P2–P15 series allowed identifying the genes undergoing development changes before and/or after the time points selected to perform HI (④,⑤ in Figure 1). Age-dependent minimum twofold changes were extracted using Volcano plot analyses and Benjamini-Hochberg unpaired *t*-test (*p* < 0.001), restricted to probes exhibiting at least twofold background on one fluorochrome (⑧ in Figure 1). Age comparisons never exceeded 1.6% false positive records at these thresholds. Age-dependent interferences of HI with development were examined relative to previous and subsequent periods (details in Supplementary Material).

Time course responses to HI were classified into 4 kinetic types. Lasting kinetics describes genes exhibiting significant regulation from 3 to 24 h post-HI. Early kinetics refers to genes regulated at 3 h post-HI that returned below detection threshold in the latest at the 24 h time point. Late kinetics includes genes detected at the 24 h time point but not earlier than 6 h post-HI. Finally, transient kinetics included genes with only regulations observed at 6 and/or 12 h post-HI.

Data in agreement with the Minimum Information on Microarray Experiment guidelines were deposited in the NCBI Gene Expression Omnibus^1^.

The data are accessible using the following accession number: GSE144456.

### Data Management

HI-induced Cy5/Cy3 mean ratio reaching statistical requirements were considered inductions or repressions depending on variation sense. Analyses of P5 and P10 induction plus repression lists were performed separately. Special attention was given to genes affected by HI at both P5 and P10 as they represented a common response to the unique insult. Genes exhibiting the same kinetics type at both ages were regrouped in a list of isochronously regulated genes.

#### Pathway Analysis

Since only 183 SeKPaths are validated in mice, we performed the analysis by two complementary approaches, using DAVID^®^ (Bioinformatics Resources 6.8, NIAID/NIH) and Ingenuity pathways analysis (IPA^®^). DAVID^®^ takes advantage of curated lists of genes while IPA^®^ is based on meta-analyses of published literature (⑨ in Figure 1).

Separate analyses were performed on P5–P10 common inductions to estimate a common core of brain responses to HI, and using restrictive threshold [number of genes (*N*) ≥ 10, *p* < 0.01 according to Benjamini corrected Fisher test and False Discovery Rate (FDR) < 10%]. Age-specific lists were analyzed in parallel. Some pathways including small series genes were considered with less than 10 records to be compared to the other age.

IPA^®^ used human gene nomenclatures (showing slight differences with mice) and did not indicate an FDR. Conversely, IPA^®^ provided a *z*-score indicating the sense of activation (inhibition) of significantly enriched (se) pathways (seIPA-Paths). We considered only canonical pathways with Bonferroni–Hochberg *p*-value < 0.001 and *N* ≥ 10 at least at a one-time point after HI. Acute-phase response signaling pathway appeared in the top significant range at all-time points and both ages. As many genes of acute-phase response contributed for high proportion to other seIPA-Paths, we restricted seIPA-Path records to those aggregating at least 5 genes after subtraction of acute phase response genes, in order to avoid over-interpretation. We also discarded some pathways related to extra-brain tissues (see complete lists in Supplementary Table 1).

#### Gene Ontology (DAVID^®^, Revigo)

Statistically enriched GO terms (SeGO-terms) were extracted using DAVID^®^ at the Bonferroni–Hochberg *p*-value < 0.001 threshold, *N* ≥ 10 and FDR < 10% from P5 or P10 gene inductions and repressions lists at every time points (⑨ in Figure 1). The lists of genes affected in common at P5 and -P10 and more strictly exhibiting isochronous kinetics were analyzed separately at the *p* < 0.01 threshold. UP_Keywords were selected at the same stringency.

Many hierarchically related and highly redundant GO terms reached statistically significant thresholds. Thus, a secondary selection was performed using Revigo to exclude redundancy (dispensability > 0.7) (Supek et al., 2011).

#### Gene Index of Regulation

The regulation of a gene at one delay post-HI and one age was based on data filtering on statistically significant inter-assay reproducible fold change. The convergences analysis using pathways and GO terms enrichments based on the sole presence criteria for each entity did not take the advantage of repeated observations (4-time points) in our protocol, the amplitude of the effects and neglected basal expression. To better consider the strength of HI effects in all entities, we calculated a regulation index (R-Index) integrating amplitude of induction (repression) at all-time points and basal expression given by Cy3 hybridization signal in naive brains.

R-Index for every gene detected at least once was calculated at one age according to the formula:

R-Index=(gene/age)(ΣsignificantFCatthe4timepoints)×logCy310

A ranking according to R-Indexes grouped in deciles was done to allow comparison of the distribution for any gene amongst deciles at P5 or P10. Secondarily the R-index rankings were used to point out the entities with major contributions in enriched pathways and GO terms.

#### Analysis of Ontogeny

Spontaneous evolution of basal expression was extracted in the two periods, P2–P10 and P5–P15, to be compared to HI induced effects at P5 and P10 respectively. Ontogenic variation interferences with HI effects were according to Pearson’s Chi^2^ test (⑩ in Figure 1).

### Protein Analyses

Protein levels were assessed on Pooled lesioned hemispheres (two of each sex) at 6 or 24 h after HI using western blot for VEGF (anti-VEGFA, Santa Cruz; sc152, 1/200), IL1-β (Santa Cruz; sc1251, 1/200) and HIF-1α (Novus Biochemicals, NB-100-479, 1/200) were investigated and were normalized on whole proteins detection since β-actin (Sigma-Aldrich, A5441, 1/1000) undergoes significant regulation under development.

A larger panel of 111 cytokines was examined by multiplex protein arrays (Mouse XL cytokine array, Proteome Profiler^TM^, R&D systems) in separate pools under manufacturer instructions. Separate pools equilibrated in sex were prepared separately (details in Supplementary Material).

## Results

### Global Analysis of HI Transcription Responses at P5 or P10

**FIGURE 2:**
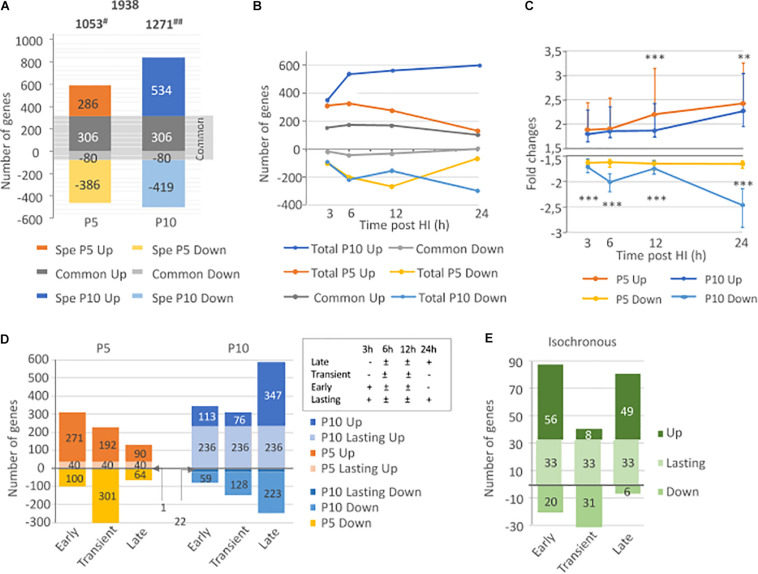
Comparison of HI effects on transcription in the neonate’s brain at P5 and P10. **(A)** Total number of inductions and repressions (noted as negative values) at P5, P10, and in common to both ages, **(B)** Time course of induction/repression numbers in P5, P10, and in common, along the 24 h period following HI **(C)** Comparison of median induction and repression amplitudes (±interquartile) along the 24 h after HI in P5 and P10 brains. **(D)** Distribution of total P5 or P10 inductions and repressions, classified as early, transient, late, or lasting kinetics (details in inset) **(E)** Kinetics distribution of genes exhibiting isochronous responses (based on the 4 classes) at both ages. ^#^Includes 5 biphasic effects (induction and repression of single genes) in P5, and ^##^ 68 biphasic regulations in P10 brains. **p < 0.01; ***p < 0.0001 according to Mann and Whitney test.

A total of 1938 genes (1053 genes at P5 and 1271 genes at P10) fulfilled the fold change (|>1.5|) and statistical (*p*-value < 0.05 in *t*-test vs. zero) criteria. In the P5 brain, HI provoked 592 gene inductions and 466 gene repressions, while in P10 brains, 840 gene inductions and 499 gene repressions were recorded. Only 386 genes (20% of the total) showed HI-induced regulations in both P5 and P10 brains (accounting for 36.7% of genes regulated at P5 and 30.4% of entities regulated at P10), and indicating that the majority of transcription effects were specific of one age (Figure 2A). Five genes at P5 and 68 genes at P10 exhibited opposite regulations at different time points after HI (biphasic responses). Detailed lists of up- and down-regulated genes after HI at P5 and/or P10, including kinetics and amplitudes, are given in Supplementary Table 1A.

Different kinetics of gene inductions were detected at P5 and P10 (see below section “Kinetics and Amplitude of HI Induced Transcription Responses at P5 or P10”). In P5 mice, the highest amplitudes were noted for genes with lasting inductions. This was neither observed at P10, nor for repression effects at both ages (Supplementary Figures 1A,B). Gene lists of HI inductions/repressions at P5 and P10 analyzed considering basal expressions, amplitudes of effects after HI, eventually, repeated observations were processed in R-index and given in Supplementary Table 1B.

The amplitudes of effects on genes affected at both ages were correlated (Pearson *r*^2^ = 0.769, *p* < 0.0001 for inductions) although only inductions account for this correlation (Supplementary Figure 1C). R-Index were also correlated (Pearson *r*^2^ = 0.704, *p* < 0.0001) (Supplementary Figure 1D). The amplitude of effects and R-Index were higher than median P5 or P10 effects, indicating that these genes affected in common at P5 and P10 may constitute a core of HI response (Supplementary Figure 1E). Only 188 genes (9.7% of total observations) had an isochronous response to HI in P5 and P10 brains (137 induction and 53 repressions, details in Supplementary Table 1C).

Only 15 genes showed opposite regulations in response to HI depending on age. Only 2 showed several records in at least 2-time points at the two ages; namely RNA methylation enzyme *Fdxacb1*, and the lymphocyte differentiation factor *Bcl11b* (Supplementary Table 1A).

Very few genes showed opposite signals on distinct probes suggesting alternative splicing; 3 genes at P5 (*Cmsd3, Crk, Epha5*), and 8 genes at P10 (*Ahnak, Dennd4a, Epha5, Kctd11, Kctd14, Plagl1, Sacs, Trim25*). Of note, transcription of *Epha5*, involved in axon guidance, showed similar transient induction of A_55_P2062911 probe and symmetrical repression of the A_55_P2100425 probe, in P5 and P10 brains (Supplementary Table 1A).

### Kinetics and Amplitude of HI Induced Transcription Responses at P5 or P10

Transcription response kinetics and amplitudes were different in P5 and P10 brains. In P5 mice, the inductions appeared monophasic, with early onset, a peak at 6 h, and a large decrease at 24 h post-HI. In P10 mice, inductions number continued to increase rapidly between 3 and 6 h, then at a slower rate until 24 h post HI. Repressions were monophasic at P5 and biphasic at P10, with a transient peak at 6 h and a strong increase in the number of genes 24 h after HI (Figure 2B). A high proportion of genes induced at P5 were also induced at P10 (more than 50% at every time point), while repression in common was scarce.

The median amplitude of inductions rose along the 24 h post-HI at both stages but with a 12 h delay in P10 mice to reach a near 2.5x fold change. Induction amplitudes were significantly higher in P5 than in P10 mice at 12 h (*p* < 0.001, according to Mann and Whitney test) and 24 h (*p* = 0.0088) post-HI. However, the highest inductions were recorded at P10 but concerned few entities. Repressions had significantly deeper amplitudes at P10 (*p* < 0.0001) and followed the biphasic kinetics of gene numbers (Figure 2C).

The distribution of gene transcription in the 4 kinetics classes was different at P5 and P10 (Figure 2D). P5 animals showed mainly early and transient inductions and transient repressions. In P10 brain early responses mainly lasted up to 24 h. Few transient effects (both directions) and a majority of late-onset effects were detected at P10. The isochronous 137 inductions and 53 repressions showed separate early and late induction and transient depression phases (Figure 2E and Supplementary Tables 1C, 2, 3).

**FIGURE 3:**
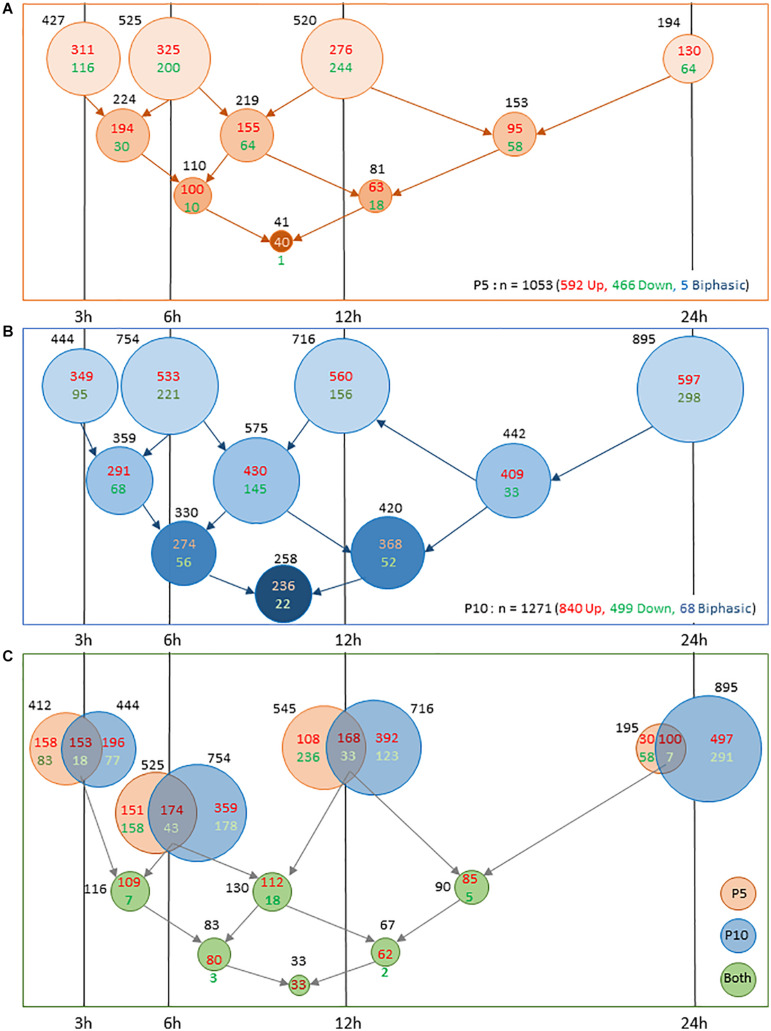
Comparison of number of genes exhibiting transcription response to HI in P5 or P10 brains at the different time intervals post HI. **(A)** Distribution in P5 mice. **(B)** Distribution in P10 mice. Lower series of circles indicate numbers of genes present in successive intervals. **(C)** Coincident transcription responses in P5 and P10 brains at 3, 6 12, and 24 h after HI. Circles overlappings indicate common responses. Bottom rows indicate numbers of common observations at consecutive time points. Induction numbers were noted in red and repressions in green. Disk surfaces were proportional to gene numbers (noted in black).

Early inductions and transient repressions predominated in P5 mice. The number of gene induction largely declined at 24 h (*n* = 195 genes), only representing 18.4% of the total inductions. Of them, 153 genes were already detected at 12 h, often with higher amplitude (*n* = 104). This pattern suggests that transcription response is in extinction as soon as 24 h post-HI in P5 mice (Figure 3A). But, this apparent monophasic induction and repression kinetics in P5 brains is misleading. Only 40 genes (3.9% of total observations) exhibited lasting induction from 3 to 24 h post-HI and a low level of redundancy over consecutive time points is clear. About 50% of entities detected at 3 or 6 h were remnant at the subsequent time point, and less than 25% were observed 12 and 24 h post-HI (Figure 3A). Repeated observation of repression at 2 consecutive time points and lasting effects were even fewer, indicating that inductions, in addition to being more numerous were also more remnant than repressions. In P5 mice only 5 genes exhibited biphasic regulation (*Adam8, Egr4, Epha5, Insig1*, and *Rtp4*) (Supplementary Table 1A).

The situation was different in P10 brains (Figure 3B). The numbers of genes affected at each time point were higher than in P5 mice (near 1.5-fold more from 3 to 12 h, and 6.6-fold at 24 h post-HI) and lasting effects were more numerous. The first wave of effects was peaking 6–12 h post-HI, a high proportion of which remained detectable at 24 h. A second wave started after 12 h and contained 188 *de novo* inductions and 265 repression 24 h after HI (Figure 3B). The total of 895 entities affected 24 h post-HI represented 70.25% of the total genes affected in the P10 brain. The 236 genes exhibiting lasting (3–24 h) induction represented 28.1% of the total induction (to be compared to 6.7% at P5). Such durability was not observed on repressions at P10. The 22 genes showing 3–24 h lasting repression, only accounted for 4.4% of total repressions. Very different to P5 brain response, HI-induced transcription at P10 showed an acceleration 24 h post-HI, suggesting an impulsion of the biological response (Figure 3C). More biphasic regulations were observed in P10 mice (*n* = 68). Of them, 53 genes exhibited profound repression at 24 h after early induction. Fifteen genes exhibited an inverse pattern; early repression followed by induction (Supplementary Table 1A).

Two main features resulted from this P5 to P10 comparative analysis of transcription after HI; (i) the very distinct time course responses; early and rapidly decreasing at P5, and more progressive and lasting effects at P10, with an intense second wave. These patterns were also observed for repressions essentially transient at P5, while delayed at P10 (Figure 2D). (ii) The occurrence of a common core of effects based on genes affected at both ages representing 20% of total observations. Core response could be dissociated in 2 phases; an early and partly lasting induction wave, and a delayed induction phase (Figure 2E).

### Gene Identity, Pathway Analyses and GO-Terms Enrichment

#### Common Core of HI Responses at P5 and P10

Complete lists of genes affected by HI at P5 and/or P10 are given in details (kinetics, R-index, sense of variation) in Supplementary Table 1C.

##### Inductions

The analysis of gene inductions with top 10 R-Index at P5 and P10 revealed a massive enrichment of genes affected in common at both ages (*n* = 68) (Supplementary Tables 1A,C). Of them, 65 were in the top-10 at the two ages. Distributions of R-Index in age-specific or common inductions showed significant divergences compared to random distribution over deciles. High R-indexes were over-represented in common genes and under-represented in age-specific inductions (Supplementary Figures 2A,B). This observation reinforces the notion of a core of common inductions at both ages as already suggested by kinetics analysis. This core included acute phase response genes (*Cebpb*, *Fos, Hmox1, Jun, Socs3, Tnfrsf1a*), as revealed by IPA^®^ analysis (see “Pathway convergences in P5 and P10 regulations”), together with some other early genes (*Egr1, Egr2, Hspb1, Ier3, Litaf, Plek*). Also detected in this core, the energy metabolite transporters (*Slc2a1* coding the glucose transporter Glut 1 and *Slc16a6* encoding the monocarboxylate transporter MCT7). Many inflammation-related genes were also present (*Ccl2, Ccl3, Ccl4, Ccl6, Ccl7, Ccl9, Ccl12, Cd14, Tnfrss1a*), as well as many transcription regulators (*Atf3, Cebpb, Ceppd, Cited1, Gadd45b, Gadd45g, Klf4, Maff, Neat1, Nr4a1*). Altogether the data pointed out an age-independent core response to HI, affecting gene transcription and inflammation. Differences in kinetics among common genes were noted for 24 items exhibiting delayed or prolonged kinetics in P10 compared to P5 brains.

##### Repressions

Fewer gene repressions than inductions were common at the two ages (*n* = 30). Of them, only 7 were in the first decile at both ages, including two genes coding energy substrate transporters (*Slc2a5*, encoding a fructose transporter, and *Slc16a7*, encoding monocarboxylate transporter MCT2 (Supplementary Figures 2C,D). It is worth noting that these convergent genes, although non-statistically validated, encoded proteins of the cell membrane, including structural components (*Nrxn3, Ntng1, Pclo, Sl40a1, Slc16a7, Slc2a5, Slitrk6, Tmsb15b1*) and signalization (*Fzd10, Gria2, Kcns3, Kcne2, P2ry12, P2ry13, Tnfaip8*). The onset of repression on these genes at P10 did not show the same delays as observed for inductions. More genes repressed were specific to P5 (*n* = 37) than to P10 (*n* = 16). Contrary to the global tendency, P10 specific repressions appeared early, and included many regulators of gene expression, while P5 specific genes had later onset (Supplementary Table 1A).

##### Data mining

**FIGURE 4:**
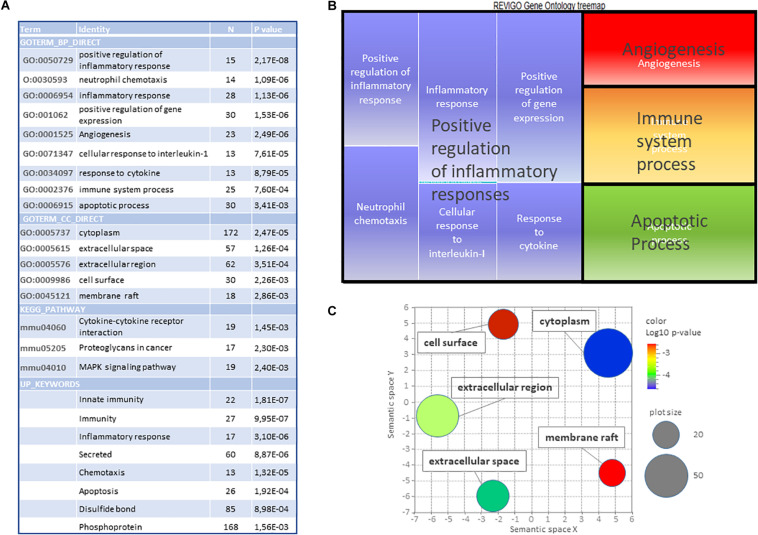
Gene ontology study of the common core of 386 genes affected by HI in P5 and P10 brains. **(A)** DAVID (v6.8) identification of significantly enriched GO-terms (dispensability < 0.7), Kegg Pathways and Keywords (*N* ≥ 10, *p* < 0.01). **(B)** Treemap representation of biological processes GO terms (BP). **(C)** Scatterplot representation of cell components (CC) GO terms. Color scale indicates *p*-values. Circle diameters represent the number of genes associated.

DAVID^®^ gene ontology (plus Revigo filtering) based on the 386 genes affected at both ages, revealed enrichment in 14 GO-terms (seGO-terms), 3 KEGG (Kyoto encyclopedia of genes and genotypes) pathways (seK-paths), and 7 Up_Keywords (Figure 4A). The analysis did not consider kinetics. Indeed, a separate analysis of isochronous genes provided very scarce information (not shown). Five main biological functions (BF) could be identified (Figure 4B), inflammation, immunity, positive regulation of gene expression, angiogenesis, and apoptosis. The same analysis performed separately on inductions revealed very similar lists (although with lower *p*-values), while the study performed on repressions revealed no enriched term (not shown). Cell component (CC) GO-terms converge to the generic term “cytoplasm” and toward cell membrane and extracellular space (Figure 4C). Separate research on P5 and P10 responses indeed confirmed enrichments in these terms (GO-terms and pathways) although including large proportions of genes induced at one age only, indicating that age-dependent responses at least in part brought age-specificity around the core response (Supplementary Material and Supplementary Tables 1D–F). Also, UP_Keywords pointed out immunity, inflammation, and secretion at high significant levels (Figure 4A).

#### Age-Dependent Transcription Effects of HI on Neonate’s Brain at P5 or P10

##### Pathway convergences in P5 and P10 regulations

Although specific gene regulations occurred according to age, common functions emerged from separate pathway analysis of P5 or P10 lists of regulations. DAVID^®^ and IPA^®^ allowed extracting 8 seKpaths and 36 seIPA-Paths (Table 1), including 290 and 366 genes, representing 27.4 and 15.4% of records at P5 and P10, respectively. Only 9 genes at P5 and 23 genes at P10 were included in both seKPaths and seIPA-Paths indicating that the two strategies were complementary. Few pathways were inhibited at P5 (*z*-score < -2), including 12.7% of the 466 repressions, and no pathway was inhibited at P10 (Supplementary Tables 1D,E).

**TABLE 1:** Pathways extracted from the series of genes affected after HI (inductions and repression) at P5 or P10.

		P5					P10					Common
Category	Term	*N*	*p*-value (time)*	Kinetics	FDR	*z*-score	*N*	*p*-value (time)	Kinetics	FDR	*z*-score	*N*
MAPK Signaling	KEGG mmu04010	28	2.01.E-9 (3 h)	Early	0.00		28	8.88E-5 (24 h)	Lasting	0.00		19
Cytokine–Cytokine receptor interaction	KEGG mmu04060	24	7.85E-5 (12 h)	Lasting	0.00		29	2.83E-3 (24 h)	Lasting	0.01		16
Role of Macrophages, Fibroblasts and Endothelial Cells in Rheumatoid Arthritis	Ingenuity Pathway	31	5.89E-6 (6 h)	Lasting			40	3.24E-5 (24 h)	Lasting			20
Neuroinflammation Signaling Pathway	Ingenuity Pathway	29	2.00E-5 (24 h)	Lasting		2.67	42	4.90E-7 (6 h)	Lasting		+ 3.30	16
Acute Phase Response Signaling	Ingenuity Pathway	20	1.74E-5 (12 h)	Lasting		+2.89	25	7.76E-7 (12 h)	Lasting		+2.31	15
Granulocyte Adhesion and Diapedesis	Ingenuity Pathway	19	1.20E-9 (12 h)	Lasting			16	1.12E-5 (24 h)	Late			13
ILK Signaling	Ingenuity Pathway	19	1.02E-4 (3 h)	Lasting			16	9.33E-4 (3 h)	Early		+3.00	11
Agranulocyte Adhesion and Diapedesis	Ingenuity Pathway	16	1.20E-9 (12 h)	Lasting			23	1.17E-7 (12 h)	Late			12
Communication between Innate and Adaptive Immune Cells	Ingenuity Pathway	11	7.41E-7 (24 h)	Lasting			16	1.12E-5 (24 h)	Lasting			9
Altered T Cell and B Cell Signaling in Rheumatoid Arthritis	Ingenuity Pathway	10	1.48E-6 (24 h)	Lasting			12	6.17E-4 (6 h)	Late			7
IL-6 Signaling	Ingenuity Pathway	19	1.17E-7 (6 h)	Early			22	1.32E-7 (3 h)	Lasting		+2.53	14
p38 MAPK Signaling	Ingenuity Pathway	15	1.15E-4 (3 h)	Early			15	2.75E-4 (6 h)	Late			7
Unfolded protein response	Ingenuity Pathway	10	1.78E-6 (3 h)	Early		+2.83	6	1.10E-4 (3 h)	Early		+2.24	5
**Synaptogenesis Signaling Pathway**	Ingenuity Pathway	15	2.45E-3 (12 h)	Transient		**−2.84**	26	2.34E-4 (6 h)	Early			5
Production of NO and ROS in Macrophages	Ingenuity Pathway	13	2.75E-5 (24 h)	Late		+2.45	18	5.62E-4 (12 h)	Late		+2.67	12
Tec Kinase Signaling	Ingenuity Pathway	6	7.24E-4 (24 h)	Late			15	4.79E-4(24 h)	Late		+3.05	5
14-3-3-mediated Signaling	Ingenuity Pathway	12	1.32E-4 (6 h)	Late		+2.23	15	3.39E-5 (6 h)	Late			7

**Category**	**Term**	***N***	***p*-value (time)**	**Kinetics**	**FDR**	***z*-score**						***N***

Hepatic Fibrosis Signaling Pathway	Ingenuity Pathway	32	1.02E-4 (12 h)	Lasting		2.68						22
Glucocorticoid Receptor Signaling	Ingenuity Pathway	24	9.55E-4 (3 h)	Early								12
Th1 and Th2 Activation Pathway	Ingenuity Pathway	13	8.13E-4 (6 h)	Early								7
IGF-1 Signaling	Ingenuity Pathway	11	2.75E-4 (3 h)	Early		+1.89						7
p53 Signaling	Ingenuity Pathway	11	1.48E-4 (3 h)	Early								7
Leukocyte Extravasation Signaling	Ingenuity Pathway	17	3.74E-4 (24 h)	Late								10

**Category**	**Term**	***N***	*p*-value (time)*	**Kinetics**	**FDR**	***z*-score**	***N***	***p*-value (time)**	**Kinetics**	**FDR**	***z*-score**	***N***

**VDR/RXR Activation**	Ingenuity Pathway	12	3.84E-4 (6 h)	Early		**−0.47**						7
**Superpathway of Cholesterol Biosynthesis**	Ingenuity Pathway	12	6.31E-12 (12 h)	Transient		**−3.37**						1
**LXR/RXR Activation**	Ingenuity Pathway	12	1.07E-5 (24 h)	Late		**−1.89**						9
**Neuroactive ligand interaction**	KEGG mmu04080	29	1.07E-7 (12h)	Late	0.00	**Repressed**						3
**Steroid biosynthesis**	KEGG mmu00100	8	9.77E-6 (12 h)	Late	0.00	**Repressed**						0
**Terpenoid backbone biosynthesis**	KEGG mmu00900	7	6.51E-5 (12h)	Late	0.07	**Repressed**						0
**Axon guidance**	KEGG mmu04360	12	5.43E-3 (12 h)	Transient	0.06	**Repressed**						2

**Category**	**Term**						***N***	***p*-value (time)**	**Kinetics**	**FDR**	***z*-score**	***N***

TNF signaling pathway	KEGG mmu04668						18	7.66E-4 (3h)	Lasting	0.00		11
HIF-1 signaling pathway	KEGG mmu04066						17	5.36E-5 (3 h)	Lasting	0.03		11
PI3K Signaling in B Lymphocytes	Ingenuity Pathway						23	2.04E-5 (3 h)	Early		+2.65	9
Sirtuin Signaling Pathway	Ingenuity Pathway						20	1.62E-4 (12 h)	Early		−1.06	15
Signaling by Rho Family GTPases	Ingenuity Pathway						26	6.03E-4 (12 h)	Late			11
B Cell Receptor Signaling	Ingenuity Pathway						24	1.82E-4 (24 h)	Late		+2.00	13
Aryl Hydrocarbon Receptor Signaling	Ingenuity Pathway						18	2.88E-5 (24 h)	Late			7
IL-12 Signaling and Production in Macrophages	Ingenuity Pathway						18	1.17E-4 (12 h)	Late			11
Death Receptor Signaling	Ingenuity Pathway						15	3.24E-4 (24 h)	Late		+2.11	8
Fcγ Receptor-mediated Phagocytosis in Macrophages and Monocytes	Ingenuity Pathway						13	2.29E-5 (24 h)	Late		+3.05	5
Natural Killer Cell Signaling	Ingenuity Pathway						12	2.82E-4 (24 h)	Late			4
Chemokine Signaling	Ingenuity Pathway						11	3.39E-4 (6 h)	Late			6
Th17 Activation Pathway	Ingenuity Pathway						10	1.41E-4 (24 h)	Late		+3.16	5

N indicates the number of genes associated to pathway observed at least once. *Lowest *p*-value recorded at the time interval indicated in parentheses. Bold characters indicate inhibited pathways.

Common functions were related to early and non-specific cellular stress responses (acute phase response, NO and ROS production, unfolded protein response), inflammation (IL-6 and ILK signaling, leukocyte adhesion and diapedesis, macrophage activities), immune responses (communication between T and B cells) and associated signaling (14.3.3 signaling, cytokine receptor interaction, MAP kinases signaling). More specifically to the nervous system, neuroinflammatory- and synaptogenesis-signaling pathways were enriched at both ages.

**FIGURE 5:**
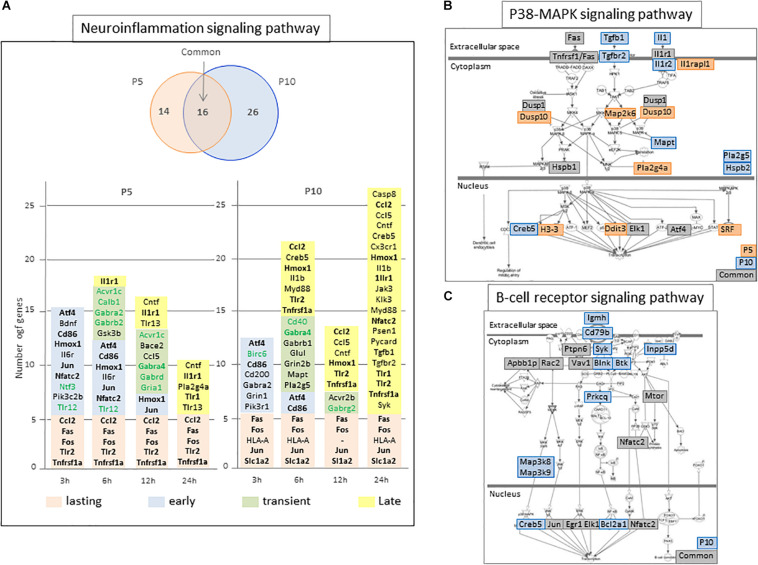
Schematic representation of selected P5 and/or P10 enriched pathways. **(A)** “Neuroinflammation signaling pathway” representation of age-specific and common numbers of genes affected in the pathway at P5 (orange circle) and/or P10 (blue circle). Genes contributing to enrichment, induced or repressed (green characters) were indicated at the different time points they were detected. Colored backgrounds refer to their regulation kinetics. Bold characters indicate genes identified in both P5 and P10 brains. **(B,C)** Schematic representation of Ingenuity pathways enriched at P5 and P10 (B; P38-MAP signaling) or P10 only (**C**; B-lymphocyte signaling). The genes detected to enrich the pathways are indicated on age dependent colored background.

The neuroinflammation pathway showed many genes in common. More than half of regulations at P5 were common with P10, while P10 specific effects predominated (Figure 5A). Genes in common mainly relate to acute phase response. Specific, inductions at P5 of *Bdnf, Cntf, Il6r*, and, *Ntf3*, as well as repressions of *Tlr12, Avcr1c, Calb1, Gabra2, Gabrb2, Gabrd*, and *Gria1*, should contribute to neuroprotection. Conversely, among regulated genes specific to P10, the induction of *Il1b, Grin1, Grin2b, Cxcr1, Casp8, Myd88* appeared potentially neurotoxic. Kinetics of neuro-inflammation pathway regulations were transient, centered 6 h after HI at P5, and biphasic at P10, with a transient activation 6 h after HI followed by a 2nd phase starting 24 h after HI (Figure 5A).

Many other convergences of pathways at the two ages were observed. Several appeared true common responses sharing more than 50% of genes (*n* = 10) and kinetics (7/10 while 3 were delayed) (Table 1). Several pathways detected at both ages had only apparent convergence. For example, the seKpath “MAPK signaling” exhibited the lowest p-values at both ages but the peak effect was delayed at P10. The seIPA-path “p38 MAPK signaling” showed less than 50% genes in common (Figure 5B).

##### Pathway divergences in P5 and P10 regulations

**FIGURE 6:**
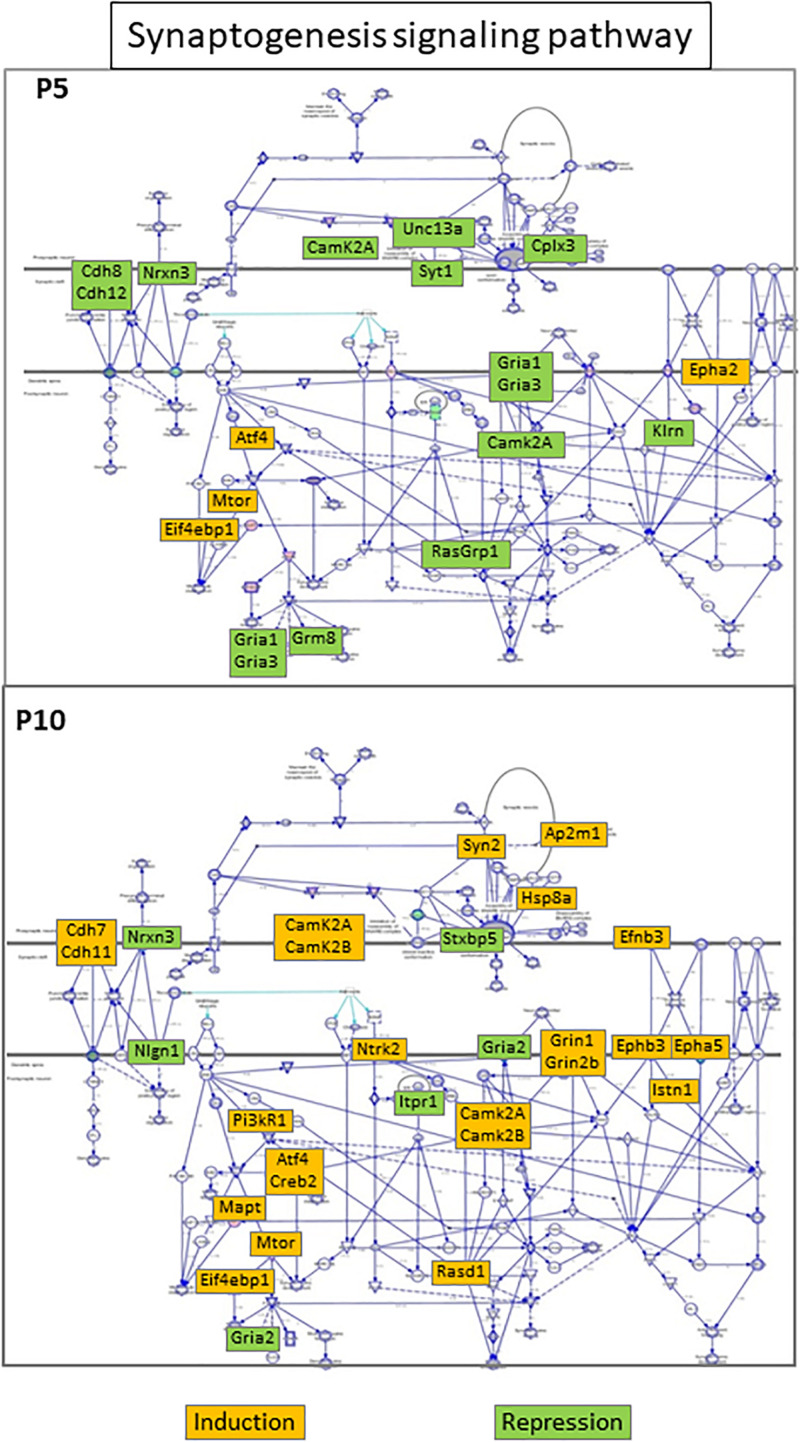
“Synaptogenesis signaling” seIPA-path schematic representation in HI exposed P5 **(upper panel)** and P10 brains **(lower panel)**. The pathway was inhibited in the P5 brain and activated in the P10 brain. Color code insets indicate the regulation direction of regulated genes.

The synaptogenesis signaling pathway significantly enriched at both ages exhibited opposite patterns (Figure 6). In the P5 brain, this pathway appeared significantly inhibited (*z*-score = −2.84, with 12/16 genes repressed) whereas it was activated at P10 (*z*-score positive but < 2). Among the 41 genes involved only 4 were detected at both ages and one (*Camk2a*) showed opposite regulations. Kinetics also was different. Most genes exhibited transient and late repression at P5, clearly not synchronous with predominant early inductions at this age. At P10, 19/26 genes in the synaptogenesis signaling pathway were activated, near half of the regulations appeared early and only 4 remained after 24 h (Eif4bp1, Mtor, Syn2, and Ntrk2), hereto not synchronous to the bulk activations at P10 (Table 1). Among the five synaptogenesis pathway associated genes in common, three were induced (*Eif4bp1, Mtor, Atf4*) and one *Nrxn3* was repressed at both ages, while Camk2A showed opposite late repression at P5 and transient activation at P10. Of note, 4 genes repressed at P5 encoded proteins involved in glutamatergic transmission (*Gria1, Gria3, Grm8, Unc13A*), while 2 essential subunits of *N*-methyl-D-aspartate glutamate receptor were enhanced at P10 (*Grin1, Grin2b*) together with many other factors involved in synaptic function (*Camk2b, Ap2m1, Creb5, Pik3r1, Syn2, Itsn1*).

##### Pathways specifically enriched in P5 brain

Thirteen seKPaths and seIPA-Paths were detected only at P5 (Table 1). The pathways based on gene inductions appeared poorly specific, sharing many genes with HI effects at P10. These pathways appeared in line with the two age common pathways, related to inflammation and immunity (hepatic fibrosis, neuroinflammation signaling, leukocyte extravasation signaling, and Th1 and Th2 activation pathways). Glucocorticoid signaling, IGF1 signaling, and P53 signaling may be considered together as protection functions and completed the series.

**FIGURE 7:**
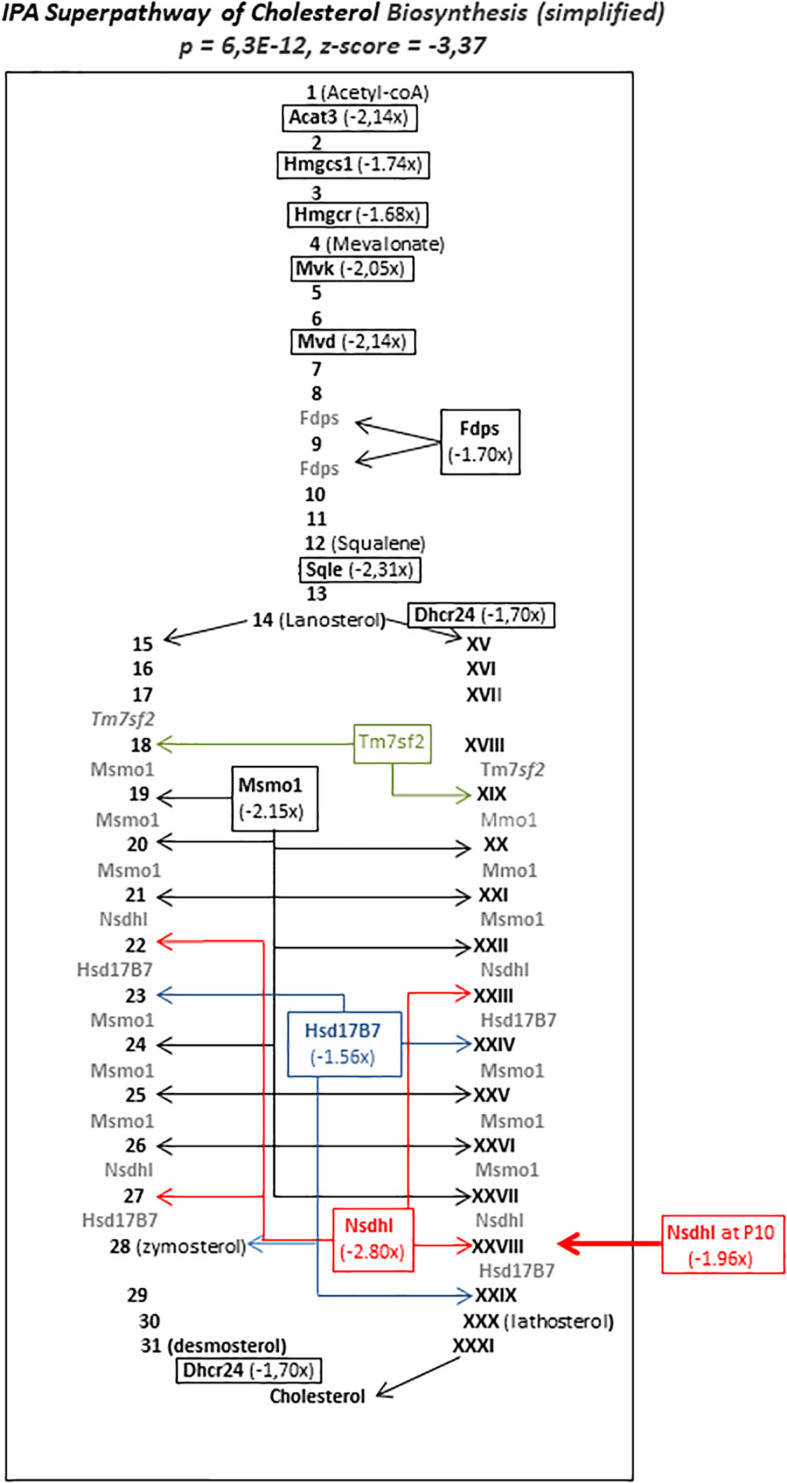
Simplified schematic representation of seIPA-path “super pathway of cholesterol biosynthesis” significantly repressed in P5 brain 12 h after HI (*p* = 6.3E-12, *z*-score = –3.37). Repressed genes figure in bold characters. Numbers (Arabic and roman) figure intermediate metabolites in two alternate biosynthesis routes. Values in parentheses indicate fold change inhibition at 12 h after HI. Acat3 enzyme coding gene (in bold italics) does not exist in Human, thus IPA^§^ release proposed the human homolog Acat2. Arrows indicate the enzyme’s multiple sites of implication. Large Arrow pointed toward Nsdhl, the only gene in this pathway affected by HI at P10.

But, the striking observation was the inhibition of the super pathway of cholesterol biosynthesis which exhibited particularly high enrichment (262-fold) and significance (*p* = 6.31E-12, *z*-score = −3.37). Twelve enzymes responsible for 2/3 of reactions in the metabolic chain were inhibited transiently 12 h after HI at P5, indicating that the inhibition of this essential metabolism is specifically affected in P5 mice (Figure 7). Only *Nsdhl* transcription was inhibited in the P10 brain. Six other pathways showed reduced activity centered 12 h after HI (Neuroactive ligand interaction, steroid biosynthesis, terpenoid backbone biosynthesis, axon guidance, and LXR/RXR activation and VDR/RXR activation pathways). Very few genes in these pathways were affected at P10 indicating the P5 specificities of repressions.

The axon guidance (KEGG_mmu04360) pathway was enriched at P5, including 12 repressions. Of which *Robo2* had R-Index in the first decile and *Epha5* exhibiting putative alternative splicing. None of these genes were included in the aforementioned synaptogenesis seIPA-Path also inhibited, although providing complement clue of P5 neurodevelopmental repression effects of HI. Only 2 genes in this path were affected at P10 (*Ntng1* was repressed and *Epha5* underwent biphasic regulation).

In the P5 brain, HI induced strong and highly specific inhibition of cholesterol metabolism, and slowed down axonal growth and synaptic differentiation, i.e., by repression of AMPA (*Gria1, Gria3*) and metabotropic (*Grm8*) glutamate receptor coding genes. Of note, *Grin1* and *Grin2b* encoding two NMDA-glutamate receptor subunits predominant in the excitotoxic phenomenon were induced at P10.

##### Pathways specifically enriched in P10 brain

Fourteen pathways were extracted from P10 gene lists. Whether this response to HI is specific to P10 brain is unclear, considering the low proportions of genes not also observed at P5, nor included in acute phase reaction pathways (Table 1). Only six of these pathways showed more than 50% of genes regulated at P10 only (Table 1). B cell receptor signaling and Pi3k signaling in B lymphocytes indicated a convergence toward B-lymphocytes (Figure 5C), but very few specific genes had high R-Index of induction (*Plcb1, Map3k8, Creb5*) or repression (*Plcb4, Itpr1*). One could suggest a tendency pointing on phospholipase-C mediated effects. The same reserve can be made for the signaling by the Rho-GTPases pathway. The series of P10-specific regulations affected essentially cell attachment and cytoskeleton protein-encoding genes; *Actg1, Actb, Cdc42ep1, Cdh5, Map3k9, Pak4, Septin3*, and *Septin9* were induced while *Limk2, Cdh11, Cdh15*, and *Cdh18* were repressed (Supplementary Tables 1D,E).

The specific P10-specific pathways TNF-, HIF- 1-, Sirtuin- or chemokine-signaling showed a large proportion of genes also regulated at P5. Several immune response associated pathways appeared more specific (B-Cell receptor- and Pi3k-signaling in B lymphocytes, Fcy mediated effects in macrophages, NK cell-signaling, and chemokine-signaling and Th17 activation) while only Th1-Th2 activation signaling appeared at P5 (Table 1). Only two had largely more genes associated with P10 than at P5; Aryl hydrocarbon receptor signaling and the near significant transcriptional misregulation in cancer (not shown) pointed out on many transcription factors inductions; *Nfia, Nfib, Nfix*, and *Rara*, while the other retinoic acid receptor Rxrg was repressed. HIF-1 signaling pathway, in line with HI context, reached significance at P10, but 11/17 genes were also affected at P5. Only one pathway at P10 was oriented toward inhibition (Sirtuin signaling pathway) although its *z*-score (−1.06) was not conclusive. Most of the genes associated with this pathway were also affected at P5 (Supplementary Tables 1D,E).

##### Upstream regulators of seIPA pathways

Why did a second phase response occur in P10 and not in P5 brains exposed to HI? First of all, the P5 very specific inhibition of cholesterol biosynthesis should be an element of response. Answers to this question should be given in analyzing the differences observed between early responses at the two ages. We looked at specific early P5 effects (lacking at P10), that could block the development of a large inflammatory response and reciprocally we explored P10 specific early responses (lacking at P5) that should be inducers of the late response.

The research of upstream factors that could produce coordinated repression at P5 pointed on 3 proteins; Apolipoprotein-E (coded by *Apoe* gene), insulin receptor (IR coded by *Insr*), and Insulin-induced gene-1 protein (coded by *Insig1*). Apoe appears as a putative upstream repressor of 16 genes exhibiting actual inhibition 12 h after HI at P5, but no *Apoe* repression appeared in our observations.

Among the 200 genes identified using IPA^®^ as putative upstream regulators of genes induced in P10-24h sample with *p*-value < 1E-3, 6 were induced early in P10 brains, and only 3 had a high R-Index (*Nflbia, Il1r1*, *Foxo1*). One gene (*Rpsa*) was repressed (FC = −1.7). Otherwise, among these 200 putative upstream regulators, 7 genes also were induced early at P5, 3 in the upper R-Index quartile (*Osmr*; FC = 3.34, *Lcn2*; FC = 3.17 and *Stat3*; FC = 1.75). These observations seemed to converge toward *Il1b*, highly and durably induced (FC = 7.56) with its receptor gene *Il1r1* (FC = 3.92), peaking at 12 h in P10 brain and unaffected at P5.

##### Gene ontology analyses in age separated regulations

**FIGURE 8:**
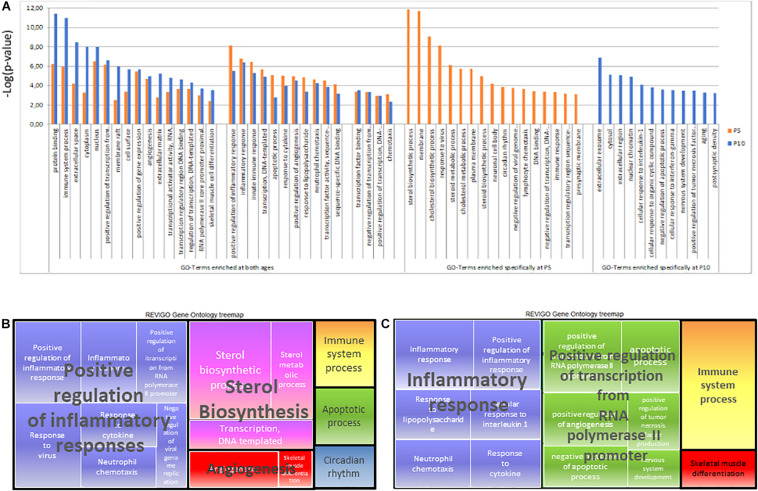
Gene ontology analysis of P5 or P10 lists of genes affected after HI. **(A)** List of terms identified using David and selected at dispensability score < 0.7 using Revigo. Enrichment significance was shown as –log(*p*-value) according to BH corrected *t*-test at P5 (orange) and P10 (blue). **(B)** Treemap of Biological Processes GOterms in P5 brain. **(C)** Treemap of Biological Processes GOterms in P10 brain.

DAVID^®^ extracted seGO-terms from P5 or P10 lists of inductions plus repressions provided large lists and high redundancies at each age (Supplementary Table 1F). More than 80% of total records were associated with seGO-terms. Analysis of all age/time points identified 60 seGO-terms (*p* < 10^–3^ according to the Bonferroni-Hochberg high stringency test and FDR < 10%). More than 50% (31/60) of seGO-terms were observed at both ages (Figure 8A). Redundancy filtering using Revigo selected 22 common terms (Table 2). Eight seGO-terms were specific to P5, mainly including the gene repressions, and six seGO-terms were enriched at P10 only, including nervous system development (23 inductions and 9 repressions) and postsynaptic density (20 inductions 6 repressions). This GO analysis revealed some differences with the pathway analysis, indicating HI effects on axon guidance and synaptogenesis specific to P5.

**TABLE 2:** GO-terms extracted from the series of genes affected after HI (Inductions and repressions) at P5 or P10, using DAVID^§^ and Revigo^§^.

				P5				P10	
				
GO-term	Description	Type	N*	*P*-value	Kinetics (*n*^#^)	*N*	*P*-value	Kinetics (*n*)	Genes in common
GO:0050729	Positive regulation of inflammatory response	BP	18	7,36E-09	Lasting	18	3,49E-06	Lasting	16
GO:0006954	Inflammatory response	BP	45	1,71E-07	Lasting	51	4,16E-07	Late (2)	28
GO:0045944	Positive regulation of transcription from RNA polymerase II promoter	BP	69	7,03E-07	Early (1)	102	2,40E-07	Early (3)	37
GO:0002376	Immune system process	BP	41	1,12E-06	Late (1)	61	1,07E-11	Late (2)	25
GO:0006351	Transcription, DNA-templated	BP	116	2,18E-06	Early (1)	164	4,04E-06	Early (2)	54
GO:0006915	Apoptotic process	BP	47	8,37E-06	Early (1)	65	2,22E-04	Lasting^$^	28
GO:0034097	Response to cytokine	BP	19	1,00E-05	Early (1)	20	1,03E-04	Late (2)	13
GO:0001525	Angiogenesis	BP	38	2,13E-05	Early (3)	38	6,94E-05	Late (2)	23
GO:0030593	Neutrophil chemotaxis	BP	15	2,41E-05	Late (2)	17	5,83E-05	Late (2)	14
GO:0071347	Cellular response to interleukin-1	BP	14	2,01E-03		18	8,49E-05	Late (2)	13
GO:0005634	Nucleus	CC	285	3,15E-07	Early (1)	634	1,06E-08	Lasting	130
GO:0005615	Extracellular space	CC	155	6,20E-05	Late (1)	136	3,43E-09	Lasting	50
GO:0009986	Cell surface	CC	64	4,18E-04	Transient (2)	74	2,09E-06	Lasting	31
GO:0005737	Cytoplasm	CC	395	5,76E-04	Early (1)	495	9,26E-09	Lasting	171
GO:0045121	Membrane raft	CC	28	3,40E-03		39	9,93E-07	Late (1)	18
GO:0005576	Extracellular region	CC	126	5,08E-03		142	8,75E-06	Late (1)	62
GO:0005515	Protein binding	MF	273	6,38E-07	Early (2)	349	3,87E-12	Lasting	124
GO:0003700	Transcription factor activity, sequence-specific DNA binding	MF	65	3,17E-05	Early (1)	82	3,50E-05	Late (1)	32
GO:0043565	Sequence-specific DNA binding	MF	46	7,00E-05	Early (1)	68	1,00E-04	Early (1)	29
GO:0044212	Transcription regulatory region DNA binding	MF	24	2,22E-04	Early (1)	30	8,00E-06	Early (1)	17
GO:0008134	Transcription factor binding	MF	30	4,22E-04	Early (1)	34	4,93E-05	Early (1)	18
GO:0001077	Transcriptional activator activity, RNA polymerase II core promoter proximal region sequence-specific binding	MF	33	5,06E-04	Early (1)	49	7,75E-06	Early (3)	19

			**P5**						

GO:0016126	Sterol biosynthetic process	BP	15	1,36E-12	Transient (1)				1
GO:0008202	Steroid metabolic process	BP	17 (1Up)	7,85E-07	Late (2)				5 (4 up)
GO:0007623	Circadian rhythm	BP	15	1,28E-04	Early (1)				9
GO:0016020	Membrane	CC	382	1,94E-12	Transient (1)				149
GO:0005886	Plasma membrane	CC	247	1,96E-06	Transient (1)				99
GO:0043025	Neuronal cell body	CC	47	6,77E-05	Transient (1)				17
GO:0042734	Presynaptic membrane	CC	12	8,40E-04	Transient (1)				3
GO:0003677	DNA binding	MF	104	3,99E-04	Early (1)				49

			**P10**						

GO:0043066	Negative regulation of apoptotic process	BP				63	3,79E-05	Early (1)	27
GO:0007399	Nervous system development	BP				34	3.32E-04	Transient (2)	13
GO:0032760	Positive regulation of tumor necrosis factor production	BP				16	3,35E-04	Late (1)	8
GO:0000790	Nuclear chromatin	CC				26	3,98E-06	Early (1)	14
GO:0005829	Cytosol	CC				160	7,09E-06	Late (3)	58
GO:0014069	Postsynaptic density	CC				26	5,85E-04	Transient (1)	5

BP indicates Gene ontology biological process classes, CC indicates Gene ontology cell component classes, MF indicates indicate Gene ontology molecular function classes. *N indicates the number of genes associated with GO-term. ^#^n indicates the number of time points of significant determination of GO-term. Lasting determination indicates enrichment at the 4-time points, ^$^apoptotic process at unique time points in the P10 brain never reached the 1E-3 threshold but reached significance when assessed over the entire period.

Six clusters of Biological Processes (BP) appeared at P5 and four at P10 (Table 2). Not surprising, major clusters were common; Inflammation, Immune system, Apoptosis angiogenesis, and regulation of transcription from RNA polymerase II promoter (exhibiting a higher weight at P10), although with kinetics differences (Supplementary Table 1F,G). The inhibition of genes of sterol metabolism had high weight and appeared very specific to P5 (GO:0016126 and GO: 0008202). Reciprocally, Response to IL-1 (GO:0071347), and TNF production (GO:0032760) were only significant at P10 and the Immune system process (GO:0002376) exhibited higher weight at P10 and nervous system development appeared also at P10 only (Figures 8B,C).

Cell component (CC) GO-terms at P5 showed a strong association with neuronal cell body and presynaptic membranes (GO:0043025 and GO:0042734) (Table 2). The reverse was observed for postsynaptic density (GO:0014069) term, indicating discrepant effects of HI at synaptic sites depending on age. At P10, more terms were associated with the nucleus, including more genes. The CC GO-term nucleus (GO:0005634) at P10, included 2.2-fold more genes (*n* = 634) than at P5 and lower *p*-value. Nuclear chromatin term (GO:0000790) appeared significantly enriched at P10 only, in line with transcription effects reported above.

On the whole, gene ontology exhibited high convergence toward inflammation, immune responses, and transcription. Although similar functions appeared, the genes involved are highly different depending on age. Sterol biosynthesis repression appeared as the major age-specific effect at P5, while the response to IL-1 and transcription regulation appeared at P10 only.

### HI Interference With Spontaneous Ontogeny at P5 or P10

A global survey of ontogenic variations around P5 (P2–P10 period) and P10 (P5–P15 period) was done to evaluate possible interferences of HI with development (Supplementary Figure 3).

**FIGURE 9:**
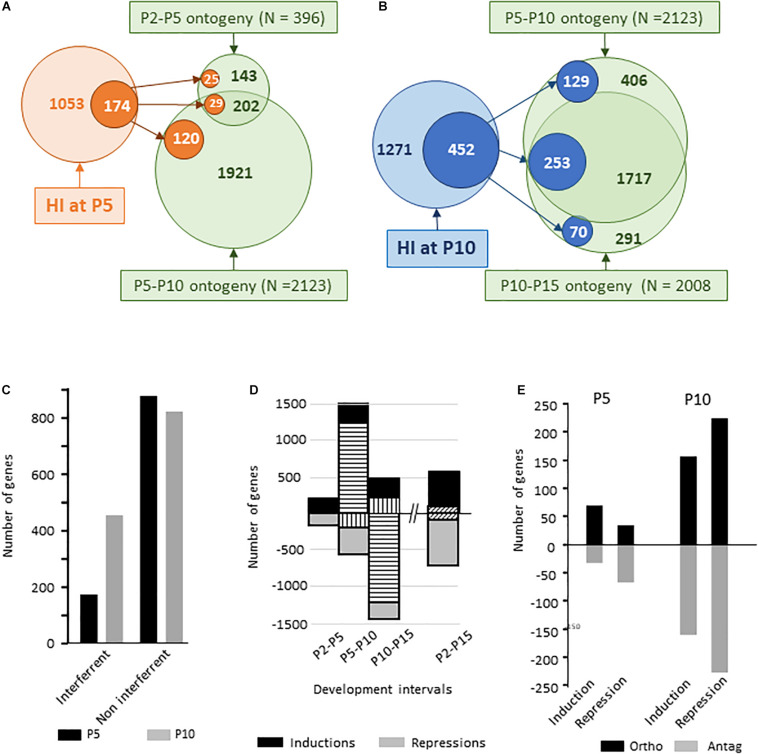
Ontogenic regulations of gene expression in dissected hemispheres over the P2–P5, P5–P10, P10–P15, and the entire P2–P15 periods. **(A)** Schematic representation of HI-evoked gene expression at P5 co-incidence with previous (P2–P5) or subsequent (P5–P10) ontogenic evolution. **(B)** Schematic representation of HI-evoked gene expression at P10 co-incidence with previous (P5–P10) or subsequent (P10–P15) ontogenic evolution. **(C)** Number of genes affected by HI at P5 (black histograms) or P10 (gray histograms) also affected along with development (co-incident) or not. HI effect on genes with spontaneous development change appeared significantly enhanced in P10 mice (χ^2^ = 354, df; 1, *p* < 0.0001). **(D)** Number of genes induced (positive values) and repressed (negative values) over the different time intervals. Horizontal hatched histograms indicate the number of genes exhibiting P5–P10 increased expression followed by P10–P15 repression. Vertical hatched histograms indicate the number of genes exhibiting P5–P10 repression followed by P10–P15 increased expression. Oblique hatching indicates gene numbers having undergone opposite regulation in the intermediate intervals and P2–P15 net variation. **(E)** Distribution of HI effect on genes with spontaneous development change according to the direction of regulation; in the direction of (ortho) or opposite (antag) to developmental direction. Distribution were statistically different at P5 and P10 (χ^2^ = 23.8, df 3, *p* < 0.0001).

Volcano plot comparisons (FC ≥ 2, *p* < 0.001) on one color hybridization signal from naive mice brain mRNAs between P2, P5, P10, and P15 showed developmental expression regulation of 6949 probes between P2 and P5, 11385 probes between P5 and P10, and 16662 probes between P10 and P15, with significant unpaired *t*-test (*p* < 0.01) (Supplementary Figure 3A). Only 4607 probes exhibited variations over the P2–P15 period resulting from the reversal of effects in consecutive time intervals and slow expression evolutions. Restriction of the analysis to probes referring to Gene Id allowed detecting developmental evolution in the transcription of 396 genes between P2 to P5, 2123 genes between P5 and P10 and 2008 genes between P10 and P15 (Figures 9A,B).

A low proportion of the genes affected by HI at P5 (16.5%, *n* = 174) concerned genes undergoing P2–P10 spontaneous evolution. This proportion was higher at P10 (35.6%, *n* = 452) relative to the P5–P15 development period (*p* < 0.0001 according to Fisher exact test) (Figure 9C). Many inductions (1281 genes) occurring between P5 and P10, were reversed between P10 to P15 period. Reciprocally 221 genes showed early repression and delayed induction (Figure 9D). The part of interaction with the series of genes exhibiting P10 centered induction and:or repression accounted for (19.7%, *n* = 253). Different types of effects were observed, i.e., inductions and repressions, in the same sense or opposite to developmental evolution. At P5, HI effects on P2–P10 regulated genes included half/half inductions and repression. HI inductions were mostly in the sense of ontogeny and repressions opposite to ontogeny (*p* < 0.001 according to Fisher exact test) (Figure 9E). HI at P5 anticipated expression of genes involved in inflammation; especially neutrophil chemotaxis, that spontaneously increased up to P10. Reciprocally, two-thirds of development antagonistic actions affected genes coding membrane-associated proteins (Supplementary Table 1H). At P10, HI evoked as much inductions as repressions on spontaneously P5–P15 regulated genes, half and half in the sense and opposite to spontaneous development (Figure 9E).

Biostatistical analysis of this development waves failed to extract GO-terms or Kegg pathways. Several generic Up_Keywords; “Disulfide bond,” “Glycoprotein,” “Signal,” and “Secreted” reached the *p*-value and FDR thresholds but with low enrichment (<1.5) (Supplementary Table 1H). Transient effects were also observed over the P2–P5 and P5–P10 consecutive periods although concerning very few entities (3 early up/late-down and 8 early down/late-up entities, not shown). Over the entire P2-P15 period, 84 genes among the 611 that globally rose had transient repression and 101 genes among the 797 genes globally repressed exhibited transient induction (Supplementary Table 1H).

### Balance Sheet of P5 and P10 Transcription Responses to HI

HI at P5 and P10 affected transcription of largely different gene sets, exhibiting distinct kinetics, although showing many convergences toward common seGO-terms and pathways and defining a common core of response. Nevertheless, age-specific effects were noted.

One may recapitulate 6 remarkable findings:

(1) Acute phase responses and inflammation processes predominate in the core of response.
(2) Convergence observed in enriched pathways and GO-terms often resulted from highly different gene sets.
(3) Kinetics of induction/repressions was very divergent between ages. P5 animals responded early after HI and tended to recover initial levels after 24 h. In P10 mice an amplification of HI effects (in gene number and amplitude of effects) was noted 24 h post-HI (and possibly lasted). Many early responsive genes at P5 showed delayed or prolonged response at P10.
(4) Gene repressions were very different. At P5 they remarkably focused on steroid metabolism and to a lesser extent to synapses. No such observation was made at P10 at gene, Pathway, or GO-term levels.
(5) IL1 signaling appeared specific to P10. Whether it is responsible for lasting and amplifying inflammation remains to be established
(6) An interference of HI with ontogeny emerged in P10 mice, but the proportion of genes with spontaneous development evolution that was affected by HI at P5 (8.2%) or P10 (18.7%) appeared finally low.

### Protein Studies

**FIGURE 10:**
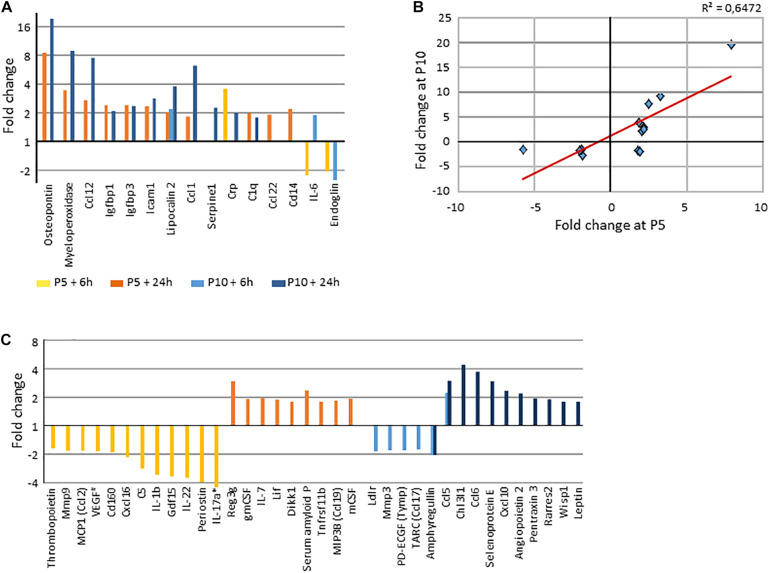
Protein array determination of HI induced variation by a minimum 1.75x in both senses. **(A)** Common proteins regulated by HI at P5 and P10 (>|1.75x|), in both senses (decreases noted as negative values). **(B)** Correlation of fold changes of proteins affected by HI at the two ages. **(C)** Age dependent regulated proteins (>|1.75x|). *, IL17a in P5 mice 6 h after HI was decrease by 8.35x, ^#^, VEGF pattern is detailed in Supplementary Material.

Protein validation of transcription observations was assessed using western blot detection of protein β-actin, VEGFA, and HIF-1α (coded by *Actb*, *Vegfa*, and *Hif1*α) (Supplementary Figure 4), and by a multiplex cytokine protein, array performed in 6h and 24 h post-HI brain extracts (Supplementary Table 1I, 4). HI induced at least 1.75-fold increases of 21 and 22 proteins at P5 and P10, respectively (Figure 10). Fifteen proteins were affected in common, roughly correlated (Figures 10A,B). Of note early decreases were noted at P5 (*n* = 14) and at P10 (*n* = 6). Increases were mostly observed 24 h after HI (Figures 10A,C). Only a few protein variations coincided with transcriptome observation.

Proteins regulated at both ages were defense proteins (myeloperoxidase, Crp, C1q), chemotactic proteins (Ccl1, Ccl12, Ccl22), adhesion and apoptosis related proteins (Icam1, Igfbp1, Igfbp3, Lcn2). Regarding proteins specifically increased at P5, one could remark several chemotactic and/or differentiation factors to monocytes and lymphocytes (Csf1, Csf2, Il7) and regulatory factors against inflammation response or promoters of tissue adaptation (Apcs, Reg3g, Lif, Tnfrsf11b). Proteins selectively increased at P10 appeared more as actors of inflammation response (Ccl5, Ccl6, Chi3l1, Cxcl10, Rarres, Sele). At the opposite, Il1b, Il17a Il22 and Cxcl6 showed early transient decrease at P5. These data did not show strict parallelism with transcription data, but they provide a convergent notion of higher inflammation response in P10 than in the P5 mouse brain.

## Discussion

### Experimental Model

Experimental HI in mice utilizing the Rice-Vannucci procedure performed 5 or 10 days post-natal has been shown a pertinent approach to produce brain lesions and behavioral defects, somehow representative of preterm and at term infant burden observed in human (Li et al., 2017; Dupré et al., 2020). HI provokes focal necrosis and tissue atrophy resulting from cell death in the core ischemic site, loss of substance in the penumbral area and at distance effects on connectivity and myelin revealed at the behavioral level (Daher et al., 2018). The study of the entire HI hemisphere provided an overview of biological responses and the use of tissue pools overcame the sex and inter-individual variability. Transcription response in insulted area and surroundings included different effects and represented only a part of the biological responses, but it was a simple way to non *a priori* estimate global physiopathological responses. Microarrays provided very sensitive access to variations of very tiny signals. This quality was also a risk to overestimate the biological importance of observations only based on amplitude variations. That is why we calculated R-Index for every gene extracted on the amplitude of responses, also including basal expression and fold change and duration of HI effect, taking advantage of the 4-time course follow up. The meaningfulness of observations was strengthened by convergences observed in filtering versus random approaches using either database of curated genes/proteins lists approved by the community of users (Kegg pathways, GOterms), or based on the literature (IPA^®^ strategy). The cross evaluation of statistically validated changes and R-Index rankings for individual genes allowed extracting from convergent paths the putative factors of the highest importance.

There are many steps between gene transcription, the function of the neo-synthetized protein, or the loss of function due to gene repression. The literature often reported discrepancies (Carlyle et al., 2017) resulting from these steps. Refined protein studies i.e., using mass spectrometry also hold in antagonistic limitations toward either sensitivity or global description. Although partial, our immune-detection for selected proteins HIF-1, VEGF-A, and broad-spectrum protein arrays focusing on cytokines and inflammation confirmed transcriptomics findings on inflammation process, i.e., a larger responses at P10 in amplitude and duration. Indeed, it did not allow extrapolations to all individual mRNA observations.

### Global Observations

The carotid unilateral ligature unbalanced blood flow in the Willis polygon and the diminished oxygen supply resulted in hypoxia-ischemia in territories including the frontoparietal cortex and underlying corpus callosum, internal and external capsule, hippocampus and variable proportions of thalamus. Energy and oxygen supplies decreased leading to defense reaction, apoptosis and necrosis in lesion core. At the level of the entire hemisphere, inductions number exceeded repressions at both ages, indicating the mobilization of energy for inductions and a possible energy sparing due to repressions. At P10 repressions appeared at random and may be considered as energy sparing, while at P5, at least a part of repressions appeared coordinated (by inhibiting cholesterol biosynthesis, see below “Convergence in the Responses to HI in 5- and 10-Day Old Mice”). The number of genes affected in our records represented 8% of the total genome and no more than 15% of the genes expressed in the period. The rough estimation (R-Index integration) of energy expanses comparisons indicates that P5 more than P10 brains had thrifty energy management, in the eventuality of energy supply defect. The recruitment P10 of the Sirtuin pathway may rely on defective management of energy balance (Meng et al., 2017). The differences in responses to HI with age were clear, even if few genes showed the opposite variation. More featuring was the fact that the majority of records corresponded to age-specific variations, however, converging toward mostly common functions. In the main groups of functions, i.e., regulation of mRNA transcription, inflammation, cell death or angiogenesis, major age differences were observed at (i) gene identity, (ii) the number of items and median amplitudes, (iii) kinetics mostly delayed for initiation and peak responses at P10, (iv) almost completely distinct repressions (92% of entities only recorded at one age), (v) opposite evolution patterns after 12h. Global biological response to HI at P5 presents an extinguishing phenomenon at the 24h time point, whereas in P10 brain, a spreading inflammation developed. These kinetic considerations, as well as energy balance estimate, support the view that management of HI insult in the P5 brain is better controlled than in P10. How these data could be paralleled with observations in human neonates? Acquired neuro/behavioral disabilities in former preterm, to a large extent depend on primary white matter injuries and myelination defect essentially resulting in primary motor disabilities (Volpe, 2009; Korzeniewski et al., 2018). In asphyxiated term infants, the damages appeared more diffuse although impacted more severely motor and above all on cognitive neurodevelopment (Pappas and Korzeniewski, 2016), as if in P5 mice and preterm infants, acquired defects were solely due to transient oligodendroglia and connectome vulnerability.

### Convergence in the Responses to HI in 5- and 10-Day Old Mice

The minority of genes showing HI-evoked inductions at the two ages are those with the highest amplitude and duration of effects, allowing considering them as a common core of HI responses in the neonate’s brain. The analysis of this restricted list showed convergence toward a few biological functions. Apoptosis and angiogenesis were not surprising responses in this hypoxo-ischemic paradigm. Inflammation was the major convergence point in enriched pathways and GO terms. The common response included; neutrophil and macrophage recruitment, cytokine, and Il-1 signaling, well documented in neonatal rats (Hagberg et al., 2015). The literature also reported the recruitment of the immune system (Li et al., 2017). Small differences appeared in kinetics such as P10 delayed inductions of leucocyte adhesion and diapedesis pathways or response to cytokine GO term. But the majority of inflammation-related pathways gathered in lasting kinetics (Table 1). The maturation of B cell differentiation and B cell receptor signaling are progressive along with development (Basha et al., 2014), and Il-10 production by B cells has been proposed as a way of resolving inflammation process in neonatal brain (Winerdal et al., 2016; Li et al., 2017). Owing to the inflammation burst and to the absence of IL-10 activation observed at P10, B cells appeared involved in P10 response, but putative anti-inflammatory function though IL10 could not be confirmed in NMRI mice brain. Not documented in the literature, the positive regulation of mRNA transcription appeared highly significant at both ages and could be included in the common response core. The function, more centered on RNA polymerase-II promoter at P10 indicates that whatever age, the cellular machinery was turned on to allow biological responses adapted to tissue stress.

Gene repressions did not contribute significantly to the common response core to HI. Altogether, these findings indicate an active mobilization of biosynthetic resources and acute reactions, largely independent of the development schedule and without coordinated energy sparing through gene repressions.

### Age-Dependent Differential Responses to HI

#### Inhibition of Cholesterol Biosynthesis Super Pathway

The transient repression centered 12 h after HI of most genes coding cholesterol biosynthesis enzymes is a major difference between P5 and P10 brain responses to HI. This pathway included a small number of enzymes but, in the very sequential succession of its steps, the high amplitude repression of two-thirds of specific enzymes coding genes, appears very unlikely as a random effect (the 6.31E-12 *p*-value was the lowest in the whole study).

What should be the consequences of transient repression of cholesterol biosynthesis? It is tempting to speculate that this inhibition takes part in the prevention of a subsequent inflammatory response as observed at P10 since the two parts of the proposition were absent in the P10 brain. Whether this effect is a benefit, is however, unlikely. Circulating cholesterol cannot cross the blood-brain barrier and its local synthesis is required (Quan et al., 2003). This takes place at a high rate in astrocytes in the developing brain, contributing to neuronal membranes and myelin sheet formation (Goritz et al., 2005; Hussain et al., 2019).

The destination of cholesterol to myelin may be associated with the frequent burden in preterm due to high vulnerability of oligodendrocyte precursors at this age (Back, 2017). In this model, MRI-scan revealed white matter alteration in P5 but not in P10 mice (Dupré et al., 2020). Myelin elaborated by oligodendrocytes contains the largest stores of brain lipids and cholesterol (Cermenati et al., 2015). The vulnerability of oligodendrocyte precursors is usually inferred due to deficiency in antioxidant mechanisms and glutamatergic transient hypersensibility (Matute et al., 2006; Back, 2017). Alteration in cholesterol metabolism may also be noxious to pre-oligodendrocytes. Mevalonate, an early intermediate in the pathway was a long time ago known to be involved in oligodendrocyte differentiation since it restored deprivation defect in culture while cholesterol did not (Langan and Volpe, 1987). More recently the mevalonate/cholesterol unbalance resulting from Hmgcr and Sqle mutations have been described in several Rett syndrome modeling (Segatto et al., 2019). In our hands, *Hmgcr* and *Hmgcs1*, the two genes encoding the enzymes upstream to mevalonate formation were repressed after HI in P5 mice, and not at P10 mice. These repressions could thus be considered, together with the particular differentiation stage of oligodendrocyte precursors at P5, as participating in the white matter alteration specifically observed at this age in mice and in human preterm neonates.

Genetic alterations of *Nsdhl* (located in chromosome X) are associated with the severe neurological CHILD syndrome, or lissencephaly in CK-syndrome (Hussain et al., 2019). As Nsdhl participates in the late steps of biosynthesis chain, one could hypothesize that lack of cholesterol itself is involved in *Nsdhl*-altered phenotypes. Indeed, accelerated cholesterol hydrolysis by specific cholesterol 24-hydroxylase in P9 mice leads to brain damages (Lu et al., 2018). Valproate treatment during pregnancy also affected cholesterol metabolism and was associated with autism spectrum disorders and oligodendrocyte decreased number in rats (Cartocci et al., 2018). Although we did not observe transcription alteration on *Cyp46a1*, encoding the valproate target enzyme, this report, in line with our observations attests that cholesterol starvation had long term detrimental effects on brain myelination and behavior.

Of note, *Nsdhl* was the only enzyme-encoding gene in this pathway to be repressed in the P10 brain indicating that cholesterol biosynthesis defects differentially affect brain development depending on ages and with different consequences. The perturbations should at least occur in two ways: an upstream effect, induced by HI in P5 mice affecting oligodendrocytes and downstream effects affecting neurons at both ages. It remains to determine whether repression of cholesterol biosynthesis actually occurs in preterm exposed to hypoxo-ischemic insults. Surprisingly very few preclinical studies and no retrospective studies of *in utero* statin expositions evaluated the long term consequences at the brain level. Statins limited preterm birth and inflammation in preeclamptic women and in the mouse (Basraon et al., 2012; Maierean et al., 2018). But some adverse effects on birth survival, body weight, neurogenesis, sensorimotor skills, and behavior have been reported (Henck et al., 1998; Driver et al., 2016). Retrospective studies of neurobehavioral capabilities in infants exposed to statins *in utero*, taking into account term of exposure appear necessary. Several other genes of lipid metabolism indeed showed repression in the P5 brain (*Acsl4, Ldlr, Stard4*, and *Apoe* as an upstream regulator; see section “Upstream Regulations”). Special attention to these metabolisms in preterm and term infants in neonatal intensive care unit would certainly be worth undertaken, as it is notable that these genes together with Hmgcr have a lifelong pivotal role in brain homeostasis, from the neonatal period to the physiopathology of Alzheimer’s disease in elderly (Leduc et al., 2016).

#### Age-Specific Effects of HI

Synaptogenesis signaling pathway was repressed after HI at P5 and activated at P10. In addition to age opposite variations, there was a very small redundancy in terms of shared genes. The seGO-term postsynaptic density was enriched at P10 including 20 inductions of which only 4 were observed at P5. In the P5 brain, synaptic structures should be compromised due to the repression of adhesion molecules (*Cdh8, Cdh12*) vesicle secretion machinery (*Cplx3, Syt1, Unc13a*), glutamate receptors and their downstream transduction chain (*Gria1, Gria3, Camk2A, RasGrp1*). The reverse was observed at P10 with induction of adhesion molecules (*Cdh7, Cdh11*), synapsin (*Syn2*), with a switch of glutamate receptor (*Gria2* repression, *Grin1, Grin2b* inductions) and activation of several downstream transduction chain factors (Figure 6). Of note, the two NMDA-receptor subunits encoded by *Grin1* and *Grin2b* are central actors of excitotoxicity (Choi, 1992; Paoletti et al., 2013). Also, *Grin2b* induction at P10 is opposite to the spontaneous switch that normally occurs in mouse brain around P5 for the benefit of *Grin2a*, an NMDA subunit devoid of pro-death activity (Liu et al., 2004; Paoletti et al., 2013). These inductions of NMDA receptors could account for microglial sustained activation, and the inflammation burst observed after 24 h at P10 (Tahraoui et al., 2001) and could contribute to P5–P10 differences observed in kinetics of response to HI after the acute-phase reaction.

Specific P10-specific pathways often showed a large proportion of genes also regulated at P5 (TNF-, HIF- 1-, Sirtuin- or chemokine-signaling). Several immune response associated pathways were observed at P10 (B Cell receptor- and Pi3k-signaling in B lymphocytes, Fcy mediated effects in macrophages, NK cell-signaling, and chemokine-signaling and Th17 activation) while only Th1-Th2 activation signaling appeared at P5 (Table 1). The aryl hydrocarbon receptor signaling associated at P10 containing many more genes than at P5 is also involved in the regulation of immunity at the transcription level (Stockinger et al., 2014). Altogether seIPA-Paths and seGO-terms BP converged to associate larger weight to immune processes recruitment at P10 than at P5.

Nevertheless at P10, Il-1 signaling revealed by the GO approach retained our attention. *Il1b* showed a P10-only robust induction (lasting 24 h and peaking at FC = 7.56), while *Il1r1* encoding its receptor was induced at both ages, and regulatory protein-coding genes *IL1rapl1* and *Ilrapl2* were repressed at both ages. Also, *Il1r2* encoding the regulating decoy receptor was induced at P10 only.

#### Upstream Regulations

The transcription of *Insr* did not appear inhibited in our data but the inhibition of IR protein activity should be associated with many of the repressions observed (*Fdps, Hmgcs1, Mvk, Mvd, Msmo1, Sqle*, *Nsdhl*) in the cholesterol biosynthesis pathway (Miao et al., 2014) as well as *Acsl4* repressed 12 h after HI at P5 (Boudina et al., 2009). Although *Insig1* exhibited early induction at both ages, it was followed by a deep inhibition at 12–24 h in P5 brain only, an effect that should be associated with downstream repressions of *Tm7f2, Dhrc24, Fdps, Hmgcs1, Mvk* and *Sqle* of cholesterol biosynthesis pathway, and other lipid metabolism-associated genes (*Acsl4, Elovl6, Stard4, and Ldlr*), that did not occur at P10 (Engelking et al., 2005; Plantier et al., 2012). The lowering of insulin could thus participate in cholesterol biosynthesis inhibition and affect brain development at specific stages. We observed the enrichment of the IGF1 signaling pathway at P5, and 7/11 of genes involved also at P10 with high amplitude and lasting effects (*Irs2, Socs3, sfr*). Indeed HI effects at transcription level alone cannot explain how glycemia and insulinemia participated in cholesterol regulation. Hypo- and hyper-glycemia have for a long time been shown to affect brain development (Efron et al., 2003; Burns et al., 2008). One could speculate that misregulated glycemia/insulinemia in preterm may affect IR and/or Insig1 activities and in turn inhibit cholesterol biosynthesis resulting in white matter flaws.

### HI Interference With Ontogenic Evolution

Ontogeny patterning of the human brain examined at exon transcription level showed rapid variations during the third trimester of gestation and lesser in early post-natal life (Kang et al., 2011). In the mouse, RNAseq studies reported major differences in alternative splicing and editing between embryos or neonates compared to adult brains (Kang et al., 2011; Dillman et al., 2013; Fertuzinhos et al., 2014). Spatio-temporal analysis of coding, and non-coding mRNA in the neocortex, including miRNA, showed postnatal rapid evolution (Fertuzinhos et al., 2014). Our development description was limited, to evaluating putative interference of HI with the genetic development program during the time window brain neonatal vulnerability. The tendency at P5 of HI to antagonize spontaneous increase of several genes coding membrane proteins potentially contributed to limiting cell interactions although appeared of marginal influence. The spontaneous transient peak expression of more than 1200 genes at P10 was the larger ontogenic feature observed. HI affected near 60% of these genes, but the uniform distribution of effects in the sense or opposite of spontaneous variations did not indicate clear cut direction. Statistical treatment of these restricted data did no more indicate definite effects. However complete lists of P10 effects pointed out nervous system development. For us, HI interference with development affected more tissue fate at the time of HI, than interfering with the genetic development program. These few, often transient effects appeared minor facing the wide effects on inflammation, i.e., on chemotaxis and of leucocytes recruitment, cell multiplication, or death, that necessarily interfered with time window dependent cell migrations or connectivity. This interpretation also fits the reported long term determination of macrophages/microglia engraving for the memory life of early events and influencing grown-up responses to subsequent episodes (Fleiss and Gressens, 2012; Li et al., 2017).

### Conclusion

The striking difference in the effects of a similar insult between the effects at P5 (modeling preterm) and P10 (modeling at term infants) is that the inflammatory process was rapidly turned off in the youngest while it burst in a second phase in older animals. This difference may sustain more severe developmental alterations in P10 brains and reinforced the idea that acquired defect at P5 essentially depend on effects on white matter, due to the restricted time window of vulnerability of oligodendrocyte precursors. Moreover, it identifies cholesterol biosynthesis pathway inhibition as a putative trigger of this etiology.

Remarkably few seGO-terms and seKPaths were related to nervous functions. But, major age differences appeared on synaptogenesis, temporized at P5, and activated at P10. Since HI direct interference with ontogenesis appeared limited in proportion, one could consider that HI effects on the outcome rather result from the action of recruited cells and their subsequent behavior.

### Perspectives

Mouse neonates provided a pertinent surrogate to human preterm at 24 PC weeks, even if mice pups did not suffer the specific burden of preterm birth (maternal factor deprivation, respiratory insufficiency, intensive care environment). Intervention in human neonates is a high-risk perspective to interfere with normal development and many preclinical clues have, to this respect, been disappointing. The present data confirmed that the control of inflammation is the priority, and identified early stages mechanisms as putative targets of anti-inflammatory strategies. Finally, it opens the perspective of a putative link between cholesterol metabolism and myelination defects at the early stages.

## Data Availability Statement

The datasets presented in this study can be found in online repositories. The names of the repository/repositories and accession number(s) can be found below: https://www.ncbi.nlm.nih.gov/geo/query/acc.cgi?acc=GSE144456, accession no: GSE144456.

## Ethics Statement

The animal study was reviewed and approved by CENOMEXA Adresse: Inserm U982, Faculté des Sciences, Mont-Saint-Aignan, France.

## Author Contributions

ND: study design, surgery, sample preparation, data mining, and redaction. CD: data mining and redaction. BL: surgery, sample preparation, data mining, and redaction. MH: sample preparation and quality control. YD: data mining. BG: team managing and redaction. SM: study design and redaction. PL: study design and supervision, data mining, and final redaction. All authors contributed to the article and approved the submitted version.

## Conflict of Interest

The authors declare that the research was conducted in the absence of any commercial or financial relationships that could be construed as a potential conflict of interest.

## Footnotes

1 https://www.ncbi.nlm.nih.gov/geo/

## Abbreviations

BF: biological functions (as gene ontology classification)
CC: cell components (as gene ontology classification)
DAVID^§^, Bioinformatics Resources 6.8: NIAID/NIH
FDR: false discovery rate
GO: gene ontology
HI: hypoxia/ischemia
IPA: ingenuity pathway analysis (IPA^§^ trademark software)
MRI: magnetic resonance imaging
NMDA: *N*-methyl -D-aspartate
PC: post conception
P5: postnatal day 5 (P2 P7, P10 P15 respectively)
R-Index: regulation index (formula in the text)
se: significantly enriched
seIPA-Path: significantly enriched IPA canonical pathway
seK-Path: significantly enriched KEGG pathway
KEGG: (Kyoto encyclopedia of genes and genotypes)
seGO-term: significantly enriched GO term.

## References

[B1] Adle-Biassette H. Golden J. A. Harding B. (2017). Developmental and perinatal brain diseases. *Handb. Clin. Neurol.* 145 51–78. 10.1016/B978-0-12-802395-2.00006-7 28987191

[B2] Back S. A. (2017). White matter injury in the preterm infant: pathology and mechanisms. *Acta Neuropathol.* 134 331–349. 10.1007/s00401-017-1718-6 28534077 PMC5973818

[B3] Basha S. Surendran N. Pichichero M. (2014). Immune responses in neonates. *Expert Rev. Clin. Immunol.* 10 1171–1184. 10.1586/1744666X.2014.942288 25088080 PMC4407563

[B4] Basraon S. K. Menon R. Makhlouf M. Longo M. Hankins G. D. Saade G. R. (2012). Can statins reduce the inflammatory response associated with preterm birth in an animal model? *Am. J. Obstet. Gynecol.* 207 e221–e227. 10.1016/j.ajog.2012.06.020 22939729

[B5] Bilbo S. D. Schwarz J. M. (2012). The immune system and developmental programming of brain and behavior. *Front. Neuroendocrinol.* 33:267–286. 10.1016/j.yfrne.2012.08.006 22982535 PMC3484177

[B6] Bolton J. L. Bilbo S. D. (2014). Developmental programming of brain and behavior by perinatal diet: focus on inflammatory mechanisms. *Dialogues Clin. Neurosci.* 16 307–320. 10.31887/dcns.2014.16.3/jbolton 25364282 PMC4214174

[B7] Boudina S. Bugger H. Sena S. O’Neill B. T. Zaha V. G. Ilkun O. (2009). Contribution of impaired myocardial insulin signaling to mitochondrial dysfunction and oxidative stress in the heart. *Circulation* 119 1272–1283. 10.1161/CIRCULATIONAHA.108.792101 19237663 PMC2739097

[B8] Burns C. M. Rutherford M. A. Boardman J. P. Cowan F. M. (2008). Patterns of cerebral injury and neurodevelopmental outcomes after symptomatic neonatal hypoglycemia. *Pediatrics* 122 65–74. 10.1542/peds.2007-2822 18595988

[B9] Carlyle B. C. Kitchen R. R. Kanyo J. E. Voss E. Z. Pletikos M. Sousa A. M. M. (2017). A multiregional proteomic survey of the postnatal human brain. *Nat. Neurosci.* 20 1787–1795. 10.1038/s41593-017-0011-2 29184206 PMC5894337

[B10] Cartocci V. Catallo M. Tempestilli M. Segatto M. Pfrieger F. W. Bronzuoli M. R. (2018). Altered brain cholesterol/isoprenoid metabolism in a rat model of autism spectrum disorders. *Neuroscience* 372 27–37. 10.1016/j.neuroscience.2017.12.053 29309878

[B11] Catale C. Gironda S. Lo Iacono L. Carola V. (2020). Microglial function in the effects of early-life stress on brain and behavioral development. *J. Clin. Med.* 9:468. 10.3390/jcm9020468 32046333 PMC7074320

[B12] Cermenati G. Mitro N. Audano M. Melcangi R. C. Crestani M. De Fabiani E. (2015). Lipids in the nervous system: from biochemistry and molecular biology to patho-physiology. *Biochim. Biophys. Acta* 1851 51–60. 10.1016/j.bbalip.2014.08.011 25150974

[B13] Choi D. W. (1992). Excitotoxic cell death. *J. Neurobiol.* 23 1261–1276. 10.1002/neu.480230915 1361523

[B14] Daher I. Le Dieu-Lugon B. Lecointre M. Dupre N. Voisin C. Leroux P. (2018). Time- and sex-dependent efficacy of magnesium sulfate to prevent behavioral impairments and cerebral damage in a mouse model of cerebral palsy. *Neurobiol. Dis.* 120 151–164. 10.1016/j.nbd.2018.08.020 30201311

[B15] Dillman A. A. Hauser D. N. Gibbs J. R. Nalls M. A. McCoy M. K. Rudenko I. N. (2013). mRNA expression, splicing and editing in the embryonic and adult mouse cerebral cortex. *Nat. Neurosci.* 16 499–506. 10.1038/nn.3332 23416452 PMC3609882

[B16] Driver A. M. Kratz L. E. Kelley R. I. Stottmann R. W. (2016). Altered cholesterol biosynthesis causes precocious neurogenesis in the developing mouse forebrain. *Neurobiol. Dis.* 91 69–82. 10.1016/j.nbd.2016.02.017 26921468 PMC4860088

[B17] Dupré N. Arabo A. Orset C. Maucotel J. Detroussel Y. Hauchecorne M. (2020). Neonatal cerebral hypoxia-ischemia in mice triggers age-dependent vascular effects and disabilities in adults; implication of tissue plasminogen activator (tPA). *Exper. Neurol.* 323:113087. 10.1016/j.expneurol.2019.113087 31697944

[B18] Efron D. South M. Volpe J. J. Inder T. (2003). Cerebral injury in association with profound iatrogenic hyperglycemia in a neonate. *Eur. J. Paediatr. Neurol.* 7 167–171. 10.1016/s1090-3798(03)00054-0 12865056

[B19] Engelking L. J. Liang G. Hammer R. E. Takaishi K. Kuriyama H. Evers B. M. (2005). Schoenheimer effect explained–feedback regulation of cholesterol synthesis in mice mediated by Insig proteins. *J. Clin. Invest.* 115 2489–2498. 10.1172/JCI25614 16100574 PMC1184040

[B20] Fertuzinhos S. Li M. Kawasawa Y. I. Ivic V. Franjic D. Singh D. (2014). Laminar and temporal expression dynamics of coding and noncoding RNAs in the mouse neocortex. *Cell Rep.* 6 938–950. 10.1016/j.celrep.2014.01.036 24561256 PMC3999901

[B21] Fleiss B. Gressens P. (2012). Tertiary mechanisms of brain damage: a new hope for treatment of cerebral palsy? *Lancet Neurol.* 11 556–566. 10.1016/S1474-4422(12)70058-3 22608669

[B22] Goritz C. Mauch D. H. Pfrieger F. W. (2005). Multiple mechanisms mediate cholesterol-induced synaptogenesis in a CNS neuron. *Mol. Cell Neurosci.* 29 190–201. 10.1016/j.mcn.2005.02.006 15911344

[B23] Hagberg H. Mallard C. Ferriero D. M. Vannucci S. J. Levison S. W. Vexler Z. S. (2015). The role of inflammation in perinatal brain injury. *Nat. Rev. Neurol.* 11 192–208. 10.1038/nrneurol.2015.13 25686754 PMC4664161

[B24] Henck J. W. Craft W. R. Black A. Colgin J. Anderson J. A. (1998). Pre- and postnatal toxicity of the HMG-CoA reductase inhibitor atorvastatin in rats. *Toxicol. Sci.* 41 88–99. 10.1006/toxs.1997.2400 9520344

[B25] Hussain G. Wang J. Rasul A. Anwar H. Imran A. Qasim M. (2019). Role of cholesterol and sphingolipids in brain development and neurological diseases. *Lipids Health Dis.* 18:26. 10.1186/s12944-019-0965-z 30683111 PMC6347843

[B26] Jacobs S. E. Berg M. Hunt R. Tarnow-Mordi W. O. Inder T. E. Davis P. G. (2013). Cooling for newborns with hypoxic ischaemic encephalopathy. *Cochrane Database Syst. Rev.* 2013:CD003311. 10.1002/14651858.CD003311.pub3 14583966

[B27] Jiang X. Nardelli J. (2016). Cellular and molecular introduction to brain development. *Neurobiol. Dis.* 92 3–17. 10.1016/j.nbd.2015.07.007 26184894 PMC4720585

[B28] Johnston M. V. (2005). Excitotoxicity in perinatal brain injury. *Brain Pathol.* 15 234–240. 10.1111/j.1750-3639.2005.tb00526.x 16196390 PMC8095755

[B29] Kang H. J. Kawasawa Y. I. Cheng F. Zhu Y. Xu X. Li M. (2011). Spatio-temporal transcriptome of the human brain. *Nature* 478 483–489. 10.1038/nature10523 22031440 PMC3566780

[B30] Korzeniewski S. J. Slaughter J. Lenski M. Haak P. Paneth N. (2018). The complex aetiology of cerebral palsy. *Nat. Rev. Neurol.* 14 528–543. 10.1038/s41582-018-0043-6 30104744

[B31] Langan T. J. Volpe J. J. (1987). Oligodendroglial differentiation in glial primary cultures: requirement for mevalonate. *J. Neurochem.* 48 1804–1808. 10.1111/j.1471-4159.1987.tb05739.x 3033149

[B32] Leduc V. Theroux L. Dea D. Dufour R. Poirier J. (2016). Effects of rs3846662 variants on HMGCR mRNA and protein levels and on markers of Alzheimer’s disease pathology. *J. Mol. Neurosci.* 58 109–119. 10.1007/s12031-015-0666-7 26541602 PMC5138059

[B33] Leroux P. Omouendze P. L. Roy V. Dourmap N. Gonzalez B. J. Brasse-Lagnel C. (2014). Age-dependent neonatal intracerebral hemorrhage in plasminogen activator inhibitor 1 knockout mice. *J. Neuropathol. Exp. Neurol.* 73 387–402. 10.1097/NEN.0000000000000062 24709679

[B34] Li B. Concepcion K. Meng X. Zhang L. (2017). Brain-immune interactions in perinatal hypoxic-ischemic brain injury. *Prog. Neurobiol.* 159 50–68. 10.1016/j.pneurobio.2017.10.006 29111451 PMC5831511

[B35] Liu X. B. Murray K. D. Jones E. G. (2004). Switching of NMDA receptor 2A and 2B subunits at thalamic and cortical synapses during early postnatal development. *J. Neurosci.* 24 8885–8895. 10.1523/JNEUROSCI.2476-04.2004 15470155 PMC6729956

[B36] Loewy J. Jaschke A. C. (2020). Mechanisms of timing, timbre, repertoire, and entrainment in neuroplasticity: mutual interplay in neonatal development. *Front. Integr. Neurosci.* 14:8. 10.3389/fnint.2020.00008 32210771 PMC7069513

[B37] Lu F. Zhu J. Guo S. Wong B. J. Chehab F. F. Ferriero D. M. (2018). Upregulation of cholesterol 24-hydroxylase following hypoxia-ischemia in neonatal mouse brain. *Pediatr. Res.* 83 1218–1227. 10.1038/pr.2018.49 29718007 PMC6019156

[B38] Maierean S. M. Mikhailidis D. P. Toth P. P. Grzesiak M. Mazidi M. Maciejewski M. (2018). The potential role of statins in preeclampsia and dyslipidemia during gestation: a narrative review. *Expert Opin. Investig. Drugs* 27 427–435. 10.1080/13543784.2018.1465927 29672173

[B39] Matute C. Domercq M. Sanchez-Gomez M. V. (2006). Glutamate-mediated glial injury: mechanisms and clinical importance. *Glia* 53 212–224. 10.1002/glia.20275 16206168

[B40] Meng X. Tan J. Li M. Song S. Miao Y. Zhang Q. (2017). Sirt1: role under the condition of ischemia/hypoxia. *Cell Mol. Neurobiol.* 37 17–28. 10.1007/s10571-016-0355-2 26971525 PMC11482061

[B41] Miao J. Haas J. T. Manthena P. Wang Y. Zhao E. Vaitheesvaran B. (2014). Hepatic insulin receptor deficiency impairs the SREBP-2 response to feeding and statins. *J Lipid Res.* 55 659–667. 10.1194/jlr.M043711 24516236 PMC3966700

[B42] Paoletti P. Bellone C. Zhou Q. (2013). NMDA receptor subunit diversity: impact on receptor properties, synaptic plasticity and disease. *Nat. Rev. Neurosci.* 14 383–400. 10.1038/nrn3504 23686171

[B43] Paolicelli R. C. Ferretti M. T. (2017). Function and dysfunction of microglia during brain development: consequences for synapses and neural circuits. *Front. Synaptic Neurosci.* 9:9. 10.3389/fnsyn.2017.00009 28539882 PMC5423952

[B44] Pappas A. Korzeniewski S. J. (2016). Long-term cognitive outcomes of birth asphyxia and the contribution of identified perinatal asphyxia to cerebral palsy. *Clin. Perinatol.* 43 559–572. 10.1016/j.clp.2016.04.012 27524454

[B45] Plantier L. Besnard V. Xu Y. Ikegami M. Wert S. E. Hunt A. N. (2012). Activation of sterol-response element-binding proteins (SREBP) in alveolar type II cells enhances lipogenesis causing pulmonary lipotoxicity. *J. Biol. Chem.* 287 10099–10114. 10.1074/jbc.M111.303669 22267724 PMC3323060

[B46] Porte B. Chatelain C. Hardouin J. Derambure C. Zerdoumi Y. Hauchecorne M. (2017a). Proteomic and transcriptomic study of brain microvessels in neonatal and adult mice. *PLoS One* 12:e0171048. 10.1371/journal.pone.0171048 28141873 PMC5283732

[B47] Porte B. Hardouin J. Zerdoumi Y. Derambure C. Hauchecorne M. Dupre N. (2017b). Major remodeling of brain microvessels during neonatal period in the mouse: a proteomic and transcriptomic study. *J. Cereb. Blood Flow Metab.* 37 495–513. 10.1177/0271678X16630557 26873886 PMC5381447

[B48] Quan G. Xie C. Dietschy J. M. Turley S. D. (2003). Ontogenesis and regulation of cholesterol metabolism in the central nervous system of the mouse. *Brain Res. Dev. Brain Res.* 146 87–98. 10.1016/j.devbrainres.2003.09.015 14643015

[B49] Rice J. E. III Vannucci R. C. Brierley J. B. (1981). The influence of immaturity on hypoxic-ischemic brain damage in the rat. *Ann. Neurol.* 9 131–141. 10.1002/ana.410090206 7235629

[B50] Rumajogee P. Bregman T. Miller S. P. Yager J. Y. Fehlings M. G. (2016). Rodent hypoxia-ischemia models for cerebral palsy research: a systematic review. *Front. Neurol.* 7:57. 10.3389/fneur.2016.00057 27199883 PMC4843764

[B51] Segatto M. Tonini C. Pfrieger F. W. Trezza V. Pallottini V. (2019). Loss of mevalonate/cholesterol homeostasis in the brain: a focus on autism spectrum disorder and rett syndrome. *Int. J. Mol. Sci.* 20:3317. 10.3390/ijms20133317 31284522 PMC6651320

[B52] Stockinger B. Di Meglio P. Gialitakis M. Duarte J. H. (2014). The aryl hydrocarbon receptor: multitasking in the immune system. *Annu. Rev. Immunol.* 32 403–432. 10.1146/annurev-immunol-032713-120245 24655296

[B53] Supek F. Bosnjak M. Skunca N. Smuc T. (2011). REVIGO summarizes and visualizes long lists of gene ontology terms. *PLoS One* 6:e21800. 10.1371/journal.pone.0021800 21789182 PMC3138752

[B54] Tahraoui S. L. Marret S. Bodenant C. Leroux P. Dommergues M. A. Evrard P. (2001). Central role of microglia in neonatal excitotoxic lesions of the murine periventricular white matter. *Brain Pathol.* 11 56–71. 10.1111/j.1750-3639.2001.tb00381.x 11145204 PMC8098534

[B55] Tuor U. I. Grewal D. (1994). Autoregulation of cerebral blood flow: influence of local brain development and postnatal age. *Am. J. Physiol.* 267 H2220–H2228. 10.1152/ajpheart.1994.267.6.H2220 7810721

[B56] Vinall J. Miller S. P. Bjornson B. H. Fitzpatrick K. P. Poskitt K. J. Brant R. (2014). Invasive procedures in preterm children: brain and cognitive development at school age. *Pediatrics* 133 412–421. 10.1542/peds.2013-1863 24534406 PMC3934331

[B57] Volpe J. J. (2009). The encephalopathy of prematurity–brain injury and impaired brain development inextricably intertwined. *Semin. Pediatr. Neurol.* 16 167–178. 10.1016/j.spen.2009.09.005 19945651 PMC2799246

[B58] Winerdal M. Winerdal M. E. Wang Y. Q. Fredholm B. B. Winqvist O. Aden U. (2016). Adenosine A1 receptors contribute to immune regulation after neonatal hypoxic ischemic brain injury. *Purinergic Signal.* 12 89–101. 10.1007/s11302-015-9482-3 26608888 PMC4749537

