# Supplementary material for: Hypoxia-Ischemia Induced Age-Dependent Gene Transcription Effects at Two Development Stages in the Neonate Mouse Brain
Source: Front Mol Neurosci. 2020 Dec 2;13:587815. doi: 10.3389/fnmol.2020.587815 (PMC7738628; doi:10.3389/fnmol.2020.587815)
Supplement: Supplementary file 1 [file Data_Sheet_1.pdf]

Supplementary Material

1. Material and methods

1.1 Design of the study

NMRI mice (Naval Medical Research Institut) purchased from Janvier (Le Genest Saint Isle, France) were housed in controlled temperature, humidity and day/night 12/12 hours cycle, with water and food *ad libitum*.

A total of 376 pups were used for the entire study. 48 sex-matched pools of RNA (n = 6 per group) were constituted at the different ages and time after insult (n = 192 pups; see details below) and 8 sex matched groups (n = 6 per group) of naïve mice at P2 and P15 (n = 48 pups; see details below) were constituted for transcriptome study. Protein study was done in separate groups of brains (n = 40 pups; see details below)

Details in HI study
4 points (3h, 6h, 12h, 24h post insult/or controls)
2 conditions (HI, Controls)
2 ages (P5, P10)
3 replicates (groups)/condition
6 pups/replicate (3 males and 3 females).
Total = 288 pups

Details in complement ontogenesis study
2 ages (P2, P15)
6 pups/replicate (3 males and 3 females)
4 replicates (groups)
Total = 48 pups

Protein study
2 ages (P5, P10)
2 conditions (HI, Controls)
6 pups/condition (3 males and 3 females) for protein arrays.
4 pups/condition (2 males and 2 females) for Western Blot
Total = 40 pups

The question of sex dependent effects of HI was not addressed in this study for the following reasons.

1) In a previous behavior study, we did not observed sex dependent HI effects in grownups (Daher et al, 2018).
2) While mice at P5 and P10 were validated stage for transposition to human preterm and term neonates (Johnston, 2005; Hagberg et al 2015, Dupré et al 2020), the correspondence was not valid for brain sexual differentiation (Le Dieu-Lugon et al, 2020).

Therefore we did not address the question of sex in this study considering that it would not have allowed to elaborate human transposition of the data.

1.2 Hypoxia-Ischemia procedure

Surgery was performed under Isoflurane anesthesia (AbbVie, Arcueil, FRANCE ; 4% induction and 2% maintenance). First, the right common carotid artery was ligated using 8-0 PROLENE^{TM} wire (ETHICON part of JOHNSON & JOHNSON MEDICAL SAS, Issy-les-Moulineaux, France). Afterwards animals were returned for 1h to their dam, and then submitted to 40 min hypoxia (O_2/N_2 ratio 8/92%). Temperature was maintained throughout surgery by a heating carpet and hypoxia was performed in a humidified and temperature controlled (36°C) device. Naive animals were used as control.

1.2 RNA sample preparation and microarray hybridization

Mice pups at each time points and the two ages were taken from at least two different litters. Thus, we compared 12 independent arrays from lesions at P5 and similarly 12 arrays from brains of animals lesioned at P10. Briefly, brains were quickly removed, sectioned on medial line and ipsilateral hemispheres were immediately frozen in liquid nitrogen. RNA extraction was performed in individual hemispheres using NucleoSpin RNA Plus extraction kit (Macherey-Nagel, Hoerdt, France) following manufacturer's instructions. Brain tissues were defrosted in 350 µl kit lysis buffer and homogeneized using ceramic beads (1.4 mm Ozyme, Montigny le Bretonneaux) for 20s at 50hz. Total RNAs extraction was performed in individual hemispheres with Nucleospin RNA plus kit® according to manufacturer instructions (Macherey-Nagel) with and extraction volume of 350µL and stored at -80°C until use. RNA quantification and quality control was done using using a Bioanalyzer 2100 (Agilent technologies) to confirm a yield ≥ 825ng, A260/A280 >1.9 (1.9-2.1) and A260/A230 >1.8 (1.8-2.5), using Nanodrop 2000c and RNA Integrity Number (RIN) values ≥ 9 (scale 1 -10. The same quantity of each individual sample was pooled and concentration set at 100ng/2.5µl for transcriptome experiment.

1.3 Transcriptome analyses

rRNA was synthesized from 100 ng total RNA and labeled using Quick Amp Labeling Kit (Agilent Technologies). A total of 825ng of rRNA (Cy3 for control and Cy5 for test) was co-hybridized on microarrays for 17 h at 65°C on Whole Mouse Genome Oligo 4x44K microarrays (G4845A, Agilent Technologies, Les Ulis, France). Raw hybridization data, evaluated on every probe 5 µm array, using Agilent DNA microarray scanner G2565CA (Agilent Technologies), were extracted and normalized using the Lowess method by Feature extraction software (Agilent), then transferred to Genespring® (GX 12.6 software,Agilent Technologies) for data processing and data mining.

Cy3-labeled naïve mice brain tRNA and Cy5-lablede ischemic brain tRNA were co-hybridized in 24 microarrays (⑤in Figure 1). All profiling of the four time points after HI at the two developmental ages were performed in three biological replicates. Cy3-labeled P2 tRNA and Cy5-labeled P15 tRNA were co-hybridized in 4 microarrays (④in Figure 1). In each array, outlier spots and those exhibiting heterogeneous signal on one color were discarded.

Only probes referring to validated genes (described known with Official Gene Symbol) were taken into account. Probes for cRIKEN sequences were excluded from analysis. In case of multiple probes from one gene, the probe exhibiting the highest fold change was used for subsequent analyses.

Interference of HI at P5 with developmentally affected genes was examined considering HI responses at all 4 times points (3h to 24h) and spontaneous development as variations detected under aforementioned criteria between P2 and P5 and between P5 and P10. We chose the P2 stage, a shorter time lapse to P5 than the P5 to P10 period to exclude earlier birth related changes. Similarly; HI effects at P10 interference with development was examined relatively spontaneaous evolution between P5 and P10 and between P10 and P15.

1.4 Protein analyses

Protein levels were assessed on lesioned hemispheres at 6h or 24h after HI and compared to naive animals using western blot VEGF (anti VEGFA, Santa Cruz; sc152, 1/200), IL1 β (Santa Cruz; sc1251, 1/200) and HIF-1α (Novus Biochemicals, NB-100-479, 1/200).

A larger panel of 111 cytokines was examinated by multiplex protein arrays (Mouse XL cytokine array, Proteome Profiler^{TM}, R&D systems) under manufacturer instructions. Protein arrays and western blot analyses were performed on sex-matched pools of extracts.

1.4.1 Immunoblot determination of VEGF, HIF-1α, IL1-ß

After SDS-polyacrylamide gel separation, proteins were bloted on polyvinyldifluoridine membranes, incubated with anti VEGFA (Santa Cruz; sc152, 1/200), IL-1β (Santa Cruz; sc1251, 1/200), HIF-1α (Novus Biochemicals, NB-100-479, 1/200) and βactin (Sigma-Aldrich, A5441, 1/1000). Gel calibration were done using the commercial marker cocktail (Page Ruler^{TM} Prestained Protein Ladder, Thermo Scientific). Revelation was performed using secondary-antibodies coupled to peroxidase and chemoluminescence detection (ECL Plus; Bio-Rad Laboratories, Marnes la Coquette, France). Whole protein detection used tryptophane residue activation on the membrane by UV illumination and analyses were done using a blot analysis system (Image Lab software version 5.2.1 build 11 from Bio-Rad Laboratories).

1.4.2 Multiplex cytokine determination

Separate pools of protein samples from X sex-matched brains were prepared for cytokine arrays (Mouse XL cytokine array, Proteome Profiler^{TM}, R&D systems) and managed following provider's instructions. Three film expositions (2 min, 10 min and 60 min) were performed to be able to quantify proteins at very different levels in saturated spots. The 60 min exposed films could be used for all quantifications. Intermembrane standardization was performed using standards inserted by the manufacturer.

Image recording was done using a Bio-Rad camera (Imagelab v 5.1) under transmission illumination (see sample image in supplementary figure 4G). Fold change was calculated as the ratio of spot intensities of HI exposed brain reported to naïve brain in the same exposition.

1.5 Data managment

1.5.1 Statistics

Comparison amplitudes of transcription response to HI in whole series of induction and repression were done by the non parametric Mann and Whitney rank comparisons test, using GraphPad software.

1.5.2 Pathway analysis

Genes of interest contributing to seGOterms enrichment were submitted separately to DAVID® to identify the mains axes of core response using GO-terms Kegg pathways and UP_Keywords. enrichment as indexes.
Several pathways not relevant to brain function were excluded from the interpretation: e.g. Aging, Chagas disease…

1.5.3 Gene Ontology analysis

Analysis performed at each age/time point identified 59 seGOterms items under the 10^{-3} p value threshold according to the Bonferroni-Hochberg high stringency test and FDR < 10%. More than 50% (31/60) seGOterms were observed at the two ages and near all the genes affected contributed to seGOterms enrichment. But only 17.6% of these genes were affected at both ages and the proportion of repressions contributing to seGOterms at P10 was reduced compared to fraction at P5 (p = 2.82E-4,

according to Fisher exact test) (Supplementary Figure 2A). The proportion of common repressions at 5 and 10 days was even lower (p = 2.91E-13 and p = 1.19E-6 *vs* proportions at P5 and P10 respectively) indicating that gene repressions induced by HI affected different targets at 5 and 10 days. Time point observation of seGOterms allowed evaluation of kinetics. As previously observed at whole gene level; seGOterms kinetics showed early response at P5 diminishing over the 24h, and biphasic early and late onset response at P10 (Supplementary Figure 2B).

2. Results

2.1 Gene ontology

Six clusters of Biological Processes (BP) appeared at P5 and four at P10 (Table 2, Figure 8B,C). i) Inflammatory response, including in common GO:0006954, GO:0045954, GO:0034097 and GO:0030593, although with kinetics differences). Differences occurred on response to IL1 (GO:0071347), and TNF production (GO:0032760) only significant at P10. ii) Immune system process (GO:0002376) exhibited similar higher weight at P10. It was detected only 3h after HI at P5, including 41 genes while it was a lasting process at P10, including 61 genes. iii) Regulation of transcription, especially RNA-polymerase II based mRNA transcription (GO:0045944) was affected early and transiently at P5, while it had early onset, lasting occurrence had finally a higher weight at P10. Few genes in common in P5 and P10 lists (27%),were associated to this GOterm. *iv*) Apoptotic process (GO:0006915) early recruited 47 genes in P5 brains, while it appeared a lasting phenomenon including 67 genes including mostly anti-apoptotic components (65 genes) in P10 brains (GO:0043066). *v*) Angiogenesis (GO:0001525) was noticed at the two age showing highly common factors but with completely distinct kinetics. *vi*) The inhibition of genes of sterol metabolism had high weight and appeared very specific to P5 (GO:0016126 and GO: 0008202). Several other classes appeared in Revigo® treemaps, although less firmly associated to GOterms; circadian rhythm at P5, skeletal muscle cell differentiation at both ages and nervous system development at P10, in relation with apoptosis (GO:0043066) and post-synaptic density (GO:0014069) (Figure 8C).

Cell component (CC) GOterms at P5 showed strong association with neuronal cell body and presynaptic membranes (GO:0043025 and GO:0042734) (Table 2). The reverse was observed for presynaptic density (GO:0014069) term, indicating discrepant effects of HI at synaptic sites depending on age, and reminding the age discrepancies noted on synaptogenesis IPA pathway (Figure 6). Membrane associated seGOterms predominated at P5 whereas more terms were associated to nucleus, including more genes at P10. The CC GO term nucleus (GO:0005634) at P10, included 2.2 fold more genes (n= 634) than at P5 and lower p value. Nuclear chromatin term (GO:0000790) appeared significantly enriched at P10 only, in line with transcription effects reported above.

2.2 P5 to P10 correlations of effects in commonly regulated genes

393 genes were induced or repressed in both P5 and P10 brains. We have looked at putative correlation between amplitude of regulations and Regulation Index (RI, that also take into account basal expression level and duration of effect) at the 2 ages. Maximum amplitude and RI of inductions appeared highly correlated (Pearson $r = 0.8769$; $p < 0.0001$, and $r = 0.8827$; $p < 0.0001$, respectively). However, no significant correlation was noted among repression max amplitude ($r = 0.08264$, $p = 0.8022$) and hardly significant among RI (0.3775 $p = 0.0011$).

2.3 P5 to P10 correlations of effects in isochronically regulated genes

137 genes showed up regulation in P5 and P10 brain with isochronic time course. Respectively 51 genes underwent repression. We have looked at putative correlation between amplitude of inductions (repression), and between the Regulation Index (RI, that also take into account basal expression level and duration of effect) at the 2 ages. Maximum amplitude and RI of inductions appeared highly correlated (Pearson $r = 0.8847$; $p < 0.0001$, and $r = 0.8564$; $p < 0.0001$, respectively). Conversely no

significant correlation was noted among repression max amplitude (r = 0.1457, p = 0.3127) and hardly significant among RI (0.2822; p = 0.0471).

2.4 Protein studies

Western blot in P5 and P10 extracts revealed that standardization of protein analyses on actin during development would include some bias (Supplementary figure 4A, B). Conversely, protein arrays standardized on amount of protein deposit and internal standardization would preclude these bias. While western blot repeatedly showed VEGF increase in P5 and P10 brains, in coherence with mRNA data, protein array only show small amplitude decrease at P5 + 6h. As it detected tiny signal, one could imagine that VEGF antibody in array had not the sensitivity of western blot antidody and failed in detecting the protein.

3. References

Daher I., Le Dieu-Lugon B., Lecointre M., Dupre N., Voisin C., Leroux P., Dourmap N., Gonzalez B.J., Marret S., Leroux-Nicollet I., Cleren C. (2018) Time- and sex-dependent efficacy of magnesium sulfate to prevent behavioral impairments and cerebral damage in a mouse model of cerebral palsy. Neurobiol Dis 120:151-164. doi:10.1016/j.nbd.2018.08.020

Hagberg H., Mallard C., Ferriero D.M., Vannucci S.J., Levison S.W., Vexler Z.S., Gressens P. (2015) The role of inflammation in perinatal brain injury. Nat Rev Neurol 11:192-208. doi:10.1038/nrneurol.2015.13

Johnston M.V. (2005) Excitotoxicity in perinatal brain injury. Brain Pathol 15:234-240. doi:10.1111/j.1750-3639.2005.tb00526.x

Le Dieu-Lugon B, Dupré N, Legouez L, Leroux P, Gonzalez BJ, Marret S, Leroux-Nicollet I, Cleren C. 2020. Why considering sexual differences is necessary when studying encephalopathy of prematurity through rodent models. Eur J Neurosci. 52:2560-2574. doi: 10.1111/ejn.14664

Supplementary figures

Supplementary Figure 1. C omparison of HI inductions/repressions amplitudes depending on kinetics of responses. **(A-B)** Median (± inter-quartiles) of fold change induction and repressions (noted as negative values) in P5 (A) and P10 (B) brains. **(C)** Correlation plot of maximum amplitudes in genes affected in common at P5 and P10. **(D)** Correlation plot of Regulation Index in genes affected in common at P5 and P10. € Comparison of medians of inductions (repressions) in whole P5 and P10 series of genes (colored) and in the restricted series of genes affected in common at the two ages (grey). *, $p < 0.05$; ***, $p < 0.001$ according to Mann and Whitney test.

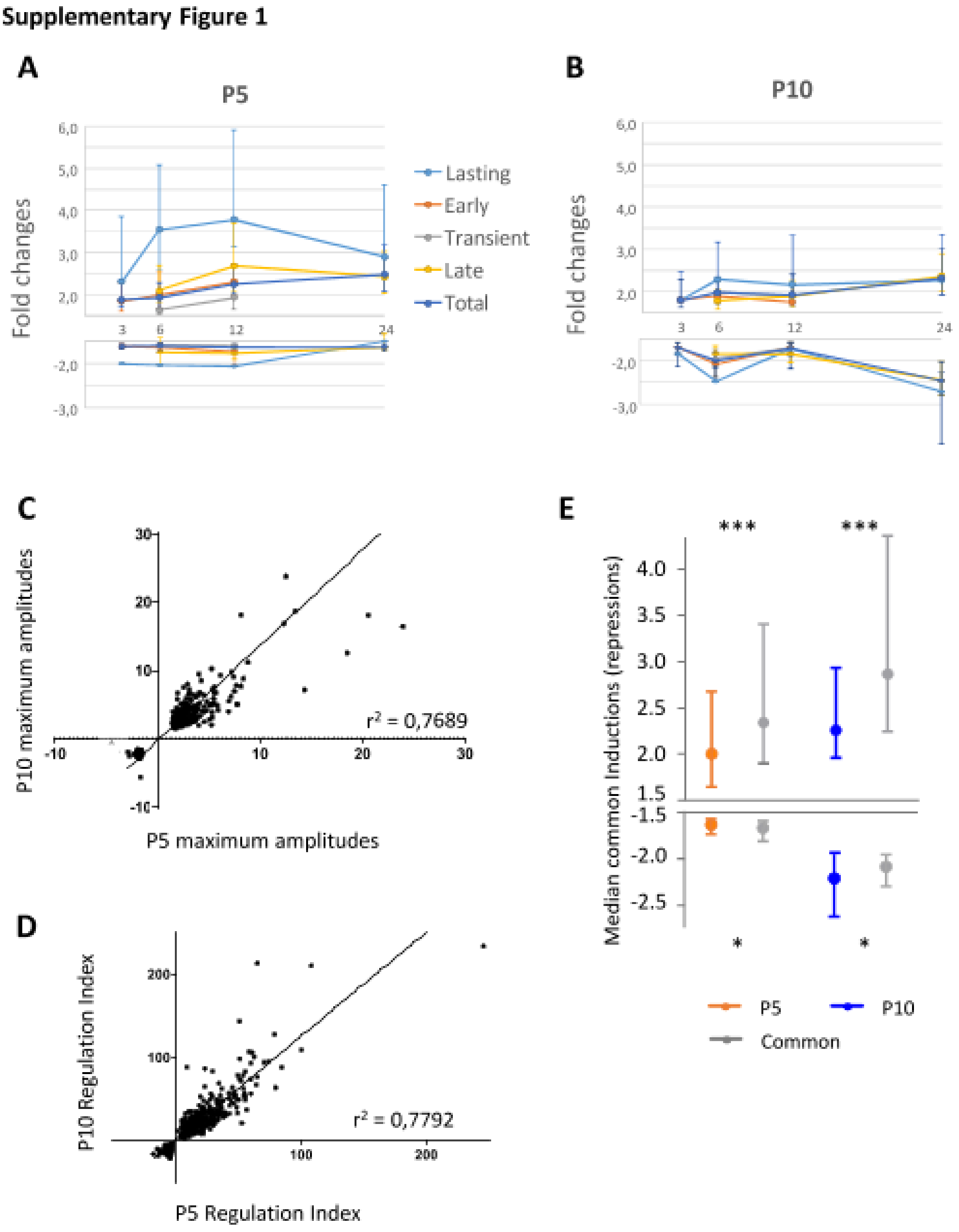

Supplementary Figure 2. Comparative distribution of age-specific and age-common effects of HI according to R-Index rankings in deciles. (**A)** P5 inductions, (**B**) P10 inductions, (**C)** P5 repressions, (**D)** P10 repressions. Inductions at P5 and P10 and repression at P10 exhibited significant enrichment in highest deciles of entities regulated in common at the 2 ages was observed amongst inductions at P5 (chi^2 = 138.5, Df 9, p <0.0001), inductions at P10 (chi^2 = 235.6, Df 9, p <0.0001), and repression at P10 (chi^2 = 119.5, Df 9, p <0.0001), but not for repressions at P5 (chi^2 = 9.863, Df 9, p = 0.3617).

Supplementary Figure 2

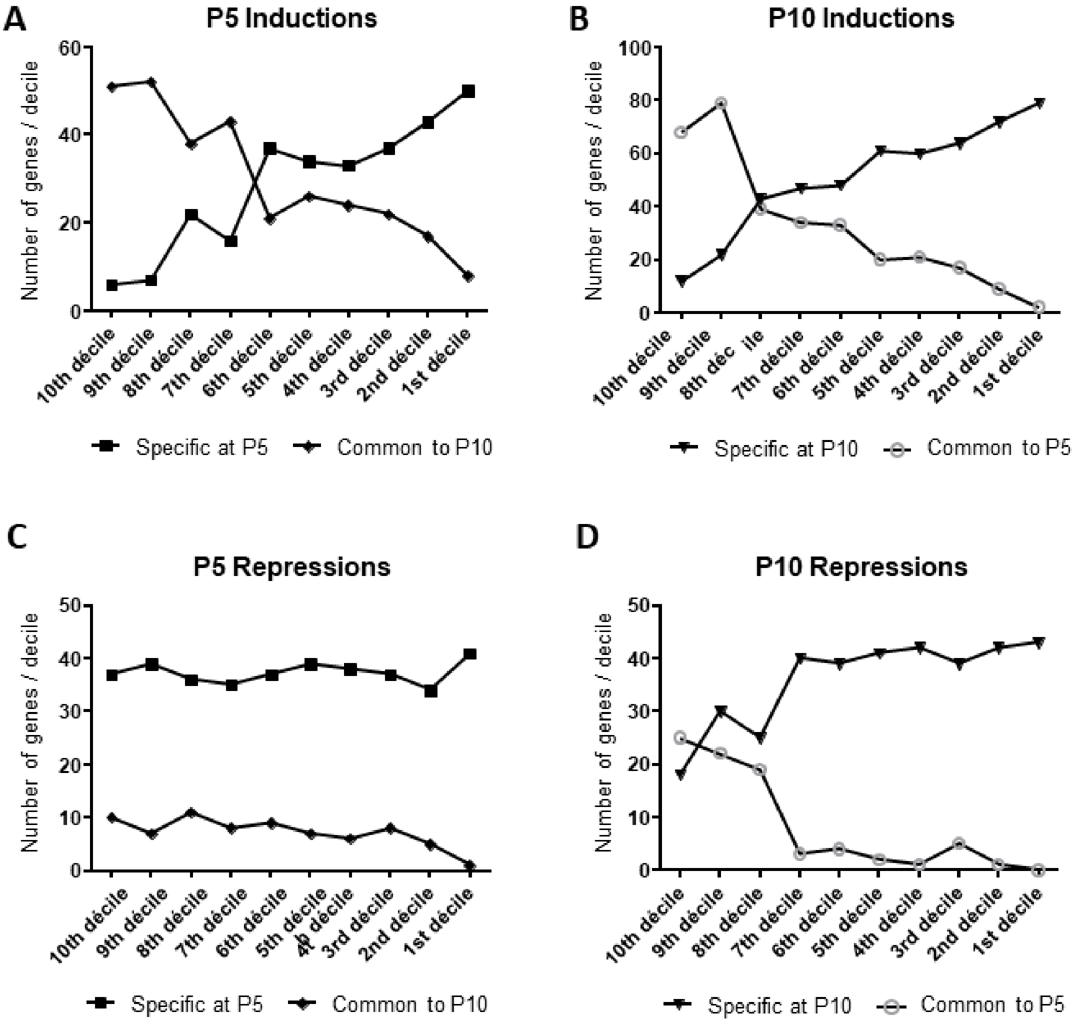

Supplementary Figure 3. Interaction of HI at P5 or P10 with developmentally regulated genes in postnatal period. (**A**) Volcano plots of spontaneous changes in gene expression during P2-P5, P5-P10, P10-P15 and P2-P15 periods (minimum amplitude 2X, p-value < 0.01 according to Bonferroni-Hochberg). (**B**) Schematic representation of HI-evoked gene expression at P5 co-incidence with previous (P2-P5) or subsequent (P5-P10) ontogenic evolution. (**C**) Schematic representation of HI-evoked gene expression at P10 co-incidence with previous (P5-P10) or subsequent (P10-P15) ontogenic evolution. (**D**). HI evoked effects in P5 mice in 4 classes of genes exhibiting distinct development regulation between P2 to P5. HI affected gene expression independently of ontogenic profiles ($Chi^2 = 7.208$, df 3, $p = 0.0656$). (**E**) HI evoked effects in P10 mice in 4 classes of genes exhibiting distinct development regulation between P5 to P15. HI affected gene expression, mainly in the opposite sense of ontogenic profiles ($Chi^2 = 320.1$, df 3, $p < 0.0001$).

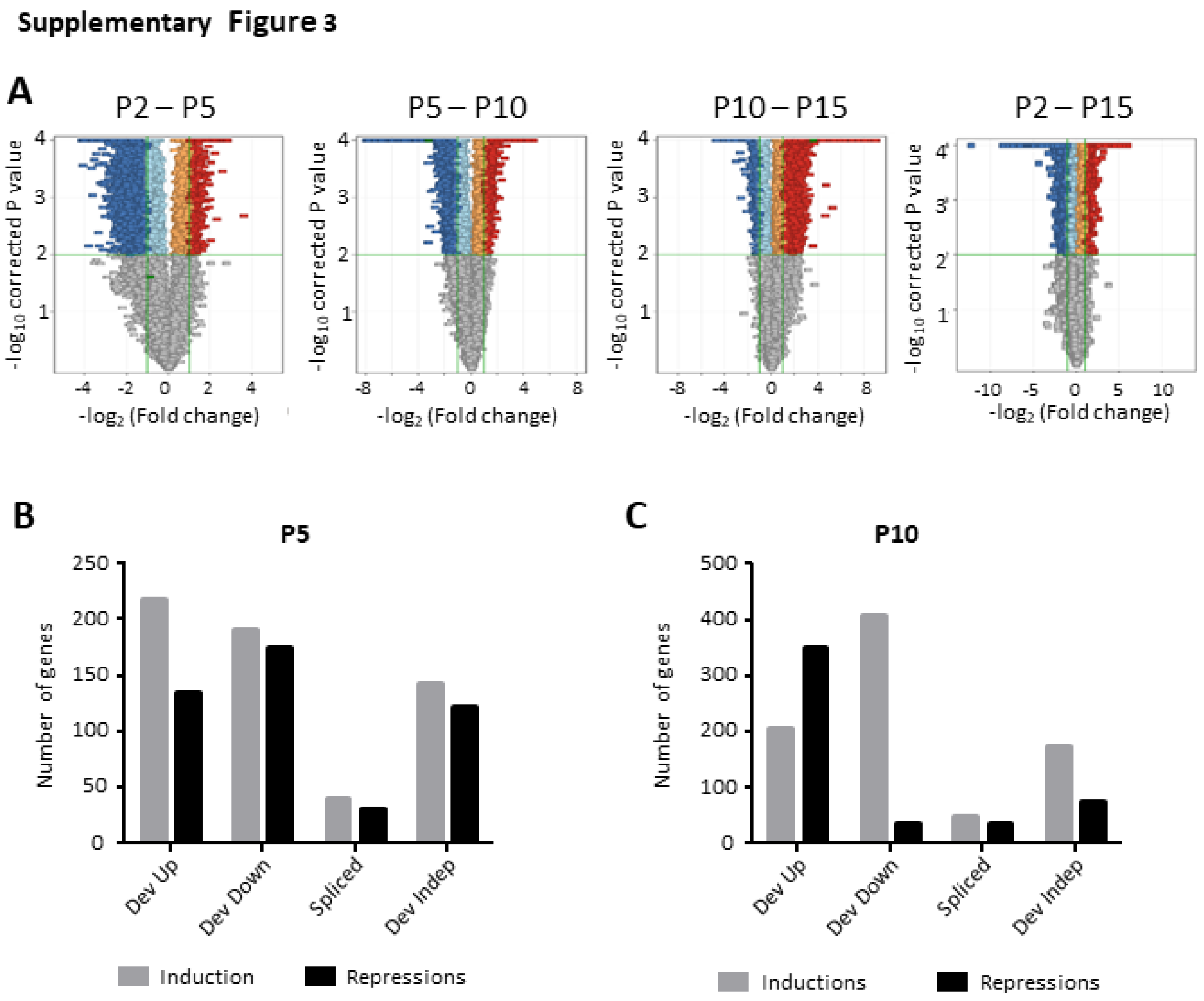

Supplementary Figure 4. Immunoblot quantitative determination of ß-actin, VEGF and HIF-1α in P5 or P10 mouse brain extracts 6 or 24h after HI. (**A**) ß-actin detection in naïve P5 and P10 mice showed significant age related decreases. (**B**) HI at P5 or P10 did not induced ßactin variation of expression. (**C**) VEGF-A monomer detection in naïve, P5 and P10 mice brains 6 and 24h. (**D**) VEGF-A-homodimer detection in the same blots as in C. (**E**) Time course evolution of VEGF monomer and dimer over 24h in P5 and P10 brains. (**F**) HIF-1α detection in naïve, P5 and P10 mice brains 6 and 24h. (**G**) Illustration of protein array detection in a P10 brain at 24h after HI (exposure 60 min). *; $p < 0.05$ according to Student't test (arrows indicate VEGF dots).

Supplementary Figure 4

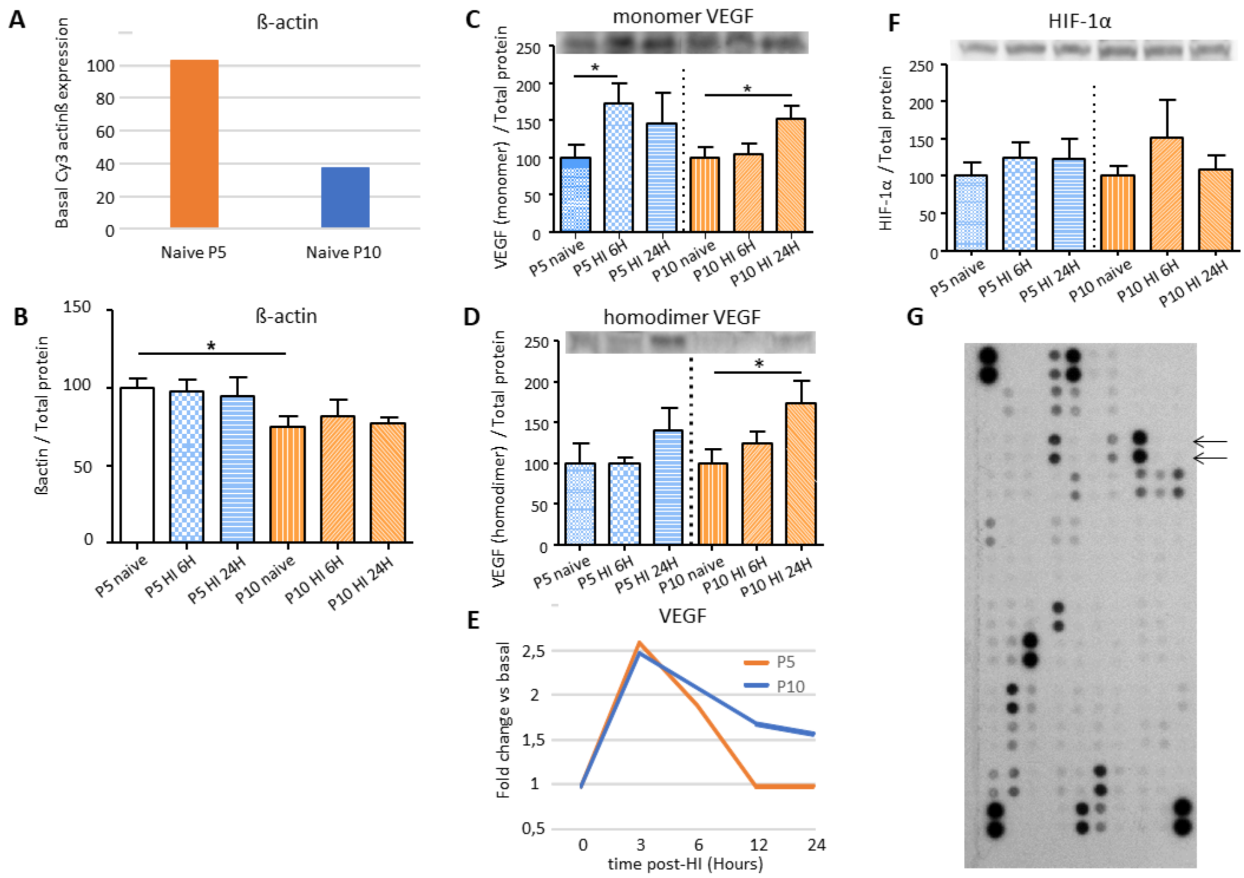

Supplementary Tables

Supplementary Table 1: Exel Table containing all steps of gene extraction and lists is provided separately (Dupre et al Supplementary Table 1.xlsx).

Supplementary Table 2. Lists of genes induced after HI at P5, P10 and both ages with regulation index in the top- 10 (in the upper decile) at least at one age. Kinetics were defined in 4 classes; Lasting; detected from 3h to 24h, Early; observed 3h after HI and returned to their basal level before 24h, Transient; observed at 6h and/or 12 h after HI only, and Late; observed at first 6h or later after HI and remaining high after 24h. Parentheses indicate an index below the top 10 at the considered age. Bold characters indicate genes with R-Index in the top-10 at both ages.

Inductions specific to P5		P5	
Gene Symbol	Gene Name	Index	Kinetic
Gdf15	growth differentiation factor 15	31	Early
Bcl3	B cell leukemia/lymphoma 3	27	Early
Hspa1a	heat shock protein 1A	27	Early
Wdr92	WD repeat domain 92	24	Early
Ecm1	Extracellular matrix protein 1	34	Transient
Lilra6	Leukocyte immunoglobulin-like receptor, subfamily A (with TM domain), member 6	60	Late

Inductions at both P5 and P10		P5		P10	
Gene Symbol	Gene Name	Index	Kinetic	Index	Kinetic
**Fos**	FBJ osteosarcoma oncogene	246	Lasting	233	Lasting
**Ccl4**	chemokine (C-C motif) ligand 4	146	Lasting	266	Lasting
**Ccl2**	chemokine (C-C motif) ligand 2	108	Lasting	210	Lasting
**Ccl12**	chemokine (C-C motif) ligand 12	100	Lasting	109	Late*
**Cebpd**	CCAAT/enhancer binding protein (C/EBP), delta	84	Lasting	88	Lasting
**Tnfrsf12a**	tumor necrosis factor receptor superfamily, member 12a	79	Lasting	63	Lasting
**Mt2**	metallothionein 2	79	Lasting	127	Lasting
**Mt1**	metallothionein 1	70	Lasting	94	Lasting
**Atf3**	activating transcription factor 3	65	Lasting	213	Lasting
**Cd14**	CD14 antigen	65	Lasting	76	Lasting
**Pip5k1a**	phosphatidylinositol-4-phosphate 5-kinase, type 1 alpha	64	Lasting	67	Lasting
**Lgals3**	lectin, galactose binding, soluble 3	62	Lasting	100	Lasting
**Ccl3**	chemokine (C-C motif) ligand 3	61	Lasting	105	Lasting
**Lilrb4**	leukocyte immunoglobulin-like receptor, subfamily B, member 4	61	Lasting	88	Lasting
**Serpina3f**	serine (or cysteine) peptidase inhibitor, clade A, member 3F	56	Lasting	37	Lasting
**Tubb6**	tubulin, beta 6 class V	55	Lasting	83	Lasting
**Ccl9**	chemokine (C-C motif) ligand 9	53	Lasting	78	Lasting
**Timp1**	tissue inhibitor of metalloproteinase 1	51	Lasting	74	Lasting
**Neat1**	nuclear paraspeckle assembly transcript 1	47	Lasting	54	Lasting
**Tuba1c**	tubulin, alpha 1C	46	Lasting	58	Lasting
**Plin2**	perilipin 2	45	Lasting	64	Lasting
**Cyp1b1**	cytochrome P450, family 1, subfamily b, polypeptide 1	43	Lasting	33	Lasting
**Ier3**	immediate early response 3	39	Lasting	33	Lasting
**Lonrf3**	LON peptidase N-terminal domain and ring finger 3	36	Lasting	62	Lasting
**Plek**	pleckstrin	34	Lasting	69	Lasting
**Tnfrsf1a**	tumor necrosis factor receptor superfamily, member 1a	33	Lasting	49	Lasting
**Maff**	v-maf musculoaponeurotic fibrosarcoma oncogene family, protein F	28	Lasting	53	Lasting
**Ctla2a**	cytotoxic T lymphocyte-associated protein 2 beta	74	Early	95	Lasting*
**Egr1**	early growth response 1	60	Early	56	Lasting*
**Jun**	jun proto-oncogene	59	Early	93	Lasting*
**Cyr61**	cysteine rich protein 61	58	Early	106	Lasting*
**Hspb1**	heat shock protein 1	55	Early	49	Lasting*
**Nr4a1**	nuclear receptor subfamily 4, group A, member 1	55	Early	53	Early
**Anxa2**	annexin A2	55	Early	57	Lasting*
Slc16a6	solute carrier family 16 (monocarboxylic acid transporters), member 6	52	Early	(21)	Early
**S100a9**	S100 calcium binding protein A9 (calgranulin B)	52	Early	71	Lasting*
**Emp1**	epithelial membrane protein 1	51	Early	98	Lasting*

**Ccl7**	chemokine (C-C motif) ligand 7	51	Early	143	Lasting*
**Ctla2b**	cytotoxic T lymphocyte-associated protein 2 alpha	50	Early	49	Early
**Cited1**	Cbp/p300-interacting transactivator with Glu/Asp-rich carboxy-terminal domain 1	49	Early	33	Early
**Gadd45b**	growth arrest and DNA-damage-inducible 45 beta	48	Early	50	Lasting*
**Angptl4**	angiopoietin-like 4	48	Early	40	Lasting*
**Gadd45g**	growth arrest and DNA-damage-inducible 45 gamma	48	Early	54	Lasting*
**Tagln2**	transgelin 2	46	Early	53	Lasting*
**Fosb**	FBJ osteosarcoma oncogene B	44	Early	47	Lasting*
**Cebpb**	CCAAT/enhancer binding protein (C/EBP), beta	42	Early	57	Lasting*
**Socs3**	suppressor of cytokine signaling 3	42	Early	50	Lasting*
**Klf4**	Kruppel-like factor 4 (gut)	37	Early	27	Early
**Btg3**	B cell translocation gene 3	37	Early	34	Early
**Litaf**	LPS-induced TN factor	37	Early	48	Lasting*
**Hmox1**	heme oxygenase (decycling) 1	36	Early	48	Lasting*
Slc2a1	solute carrier family 2 (facilitated glucose transporter), member 1	35	Early	54	Lasting*
**Slc10a1**	solute carrier family 10 (sodium/bile acid cotransporter family), member 1	32	Early	49	Early
**S100a8**	S100 calcium binding protein A8 (calgranulin A)	27	Early	56	Lasting*
**Egr2**	early growth response 2	27	Early	49	Lasting*
Jund	jun D proto-oncogene	(22)	Early	48	Lasting*
**Rnu1b6**	U1b6 small nuclear RNA	29	Transient	83	Late
**Tgm2**	transglutaminase 2, C polypeptide	23	Transient	65	Lasting*
**Ahnak**	AHNAK nucleoprotein (desmoyokin)	59	Late	73	Lasting
**Gpr84**	G protein-coupled receptor 84	54	Late	54	Late
**Spp1**	secreted phosphoprotein 1	54	Late	63	Lasting
**Vim**	vimentin	49	Late	78	Lasting
**Gfap**	glial fibrillary acidic protein	46	Late	45	Late
**Ccl6**	chemokine (C-C motif) ligand 6	42	Late	42	Late
**S100a4**	S100 calcium binding protein A4	40	Late	31	Late
**S100a6**	S100 calcium binding protein A6 (calcyclin)	38	Late	30	Late
**Ifi202b**	interferon activated gene 202B	23	Late	86	Lasting
Ctsz	cathepsin Z	(19)	Late	53	Lasting

Inductions specific to P10		P10	
Gene Symbol	Gene Name	Index	Kinetic
Actb	actin, beta	36	Lasting
Actg1	actin, gamma, cytoplasmic 1	28	Lasting
Ctsb	cathepsin B	30	Lasting
Dpysl3	dihydropyrimidinase-like 3	29	Lasting
Eno1	enolase 1, alpha non-neuron	26	Lasting
Saa3	serum amyloid A 3	67	Lasting
Stfa2l1	*cysteine-type endopeptidase inhibitor activity*[#]	38	Lasting
Glrx2	glutaredoxin 2 (thioltransferase)	30	Transient
Rn4,5s	4,5S RNA	28	Transient
ND2	NADH dehydrogenase subunit 2	26	Late
ND4L	NADH dehydrogenase subunit 4L	26	Late
Ndor1	NADPH-dependent diflavin oxidoreductase 1	26	Biphasic

Footnotes Supplementary Table 2

Index amplitudes varied from 1.5 to 246 in the 593 inductions recorded at P5 and from 1.5 to 424 in the 840 inductions recorded at P10. Bold characters indicate genes exhibiting R-index in the 1rst decile at the 2 ages. *; delayed regulation at P10, [#]*; molecular function for MGC130173 submitted name.*

Supplementary Table 3. Lists of genes repressed after HI at P5 and/or P10 with regulation index in the lowest decile (highest negative amplitude) at least at one age. Kinetics were defined in 4 classes: Lasting; detected from 3h to 24h, Early; observed 3h after HI and returned to basal level before 24h, Transient; observed at 6h and/or 12 h after HI only, and Late; observed at first 6h or later after HI and remaining high after 24h. Parentheses indicate index below the top 10 at the considered age. Bold characters indicate genes with RI in the first decile at both ages.

Repressions specific to P5		P5	
Gene Symbol	Gene Name	Index	Kinetics
Lrrc55	leucine rich repeat containing 55	-18	Early
Slc22a8	solute carrier family 22 (organic anion transporter), member 8	-13	Early
Actg2	actin, gamma 2, smooth muscle, enteric	-11	Early
Tlr12	toll-like receptor 12	-11	Early
Bcl11b	B cell leukemia/lymphoma 11B	-10	Early
Tubb4b	tubulin, beta 4B class IVB	-14	Transient
Mvd	mevalonate (diphospho) decarboxylase	-12	Transient
Grm7	glutamate receptor, metabotropic 7	-12	Transient
Robo2	Roundabout homolog 2	-12	Transient
Elovl6	ELOVL family member 6, elongation of long chain fatty acids	-11	Transient
Erbb4	v-erb-a erythroblastic leukemia viral oncogene homolog 4	-11	Transient
Fam19a1	family with sequence similarity 19, member A1	-10	Transient
Palm2	paralemmin 2	-10	Transient
Tmem74b	transmembrane protein 74B	-10	Transient
Tubb3	tubulin, beta 3 class III	-25	Late
Tubb5	tubulin, beta 5 class I	-25	Late
Sqle	squalene epoxidase	-23	Late
Msmo1	methylsterol monoxygenase 1	-21	Late
Tuba1a	tubulin, alpha 1A	-19	Late
Tuba4a	tubulin, alpha 4A	-16	Late
Hmgcs1	3-hydroxy-3-methylglutaryl-Coenzyme A synthase 1	-16	Late
Idi1	Isopentenyl-diphosphate Delta-isomerase 1	-15	Late
Mvk	mevalonate kinase	-15	Late
Btbd9	BTB (POZ) domain containing 9	-15	Late
Ldlr	low density lipoprotein receptor	-14	Late
Oxct1	3-oxoacid CoA transferase 1	-14	Late
Fdps	farnesyl diphosphate synthetase	-13	Late
Itpka	nositol-trisphosphate 3-kinase A	-11	Late
Tm7sf2	transmembrane 7 superfamily member 2	-11	Late
Kcnh5	Potassium voltage-gated channel subfamily H member 5	-11	Late
Kcnb2	Potassium voltage-gated channel subfamily B member 2	-11	Late
Gabrd	gamma-aminobutyric acid (GABA) A receptor, subunit delta	-11	Late
Zcchc16	zinc finger, CCHC domain containing 16	-10	Late
Egr4	early growth response 4	-10	Late
Slc6a4	solute carrier family 6 (neurotransmitter transporter, serotonin), member 4	-10	Late
Idh1	isocitrate dehydrogenase 1 (NADP+), soluble	-10	Late
Crhr1	corticotropin releasing hormone receptor 1	-10	Late

Repressions at both P5 and P10		P5		P10	
Gene Symbol	Gene Name	Index	Kinetic	Index	Kinetic
**Tmsb15b1**	thymosin beta 15b1	-14	Lasting	-18	Late
**Kcns3**	potassium voltage-gated channel, delayed-rectifier, subfamily S, member 3	-12	Early	-14	Early
P2ry13	purinergic receptor P2Y, G-protein coupled 13	-12	Early	-12	Early
**Slc16a7**	solute carrier family 16 (monocarboxylic acid transporters), member 7	-13	Transient	-13	Transient
**Nrxn3**	neurexin III	-12	Transient	-14	Transient
**Izumo4**	IZUMO family member 4	-10	Transient	-15	Late
**P2ry12**	purinergic receptor P2Y, G-protein coupled 12	-18	Late	-16	Late
Anks1b	ankyrin repeat and sterile alpha motif domain containing 1B	-15	Late	(-10)	Transient

Nsdhl	NAD(P) dependent steroid dehydrogenase-like	-14	Late	(-11)	Late
**Slc2a5**	solute carrier family 2 (facilitated glucose transporter) member 5	-13	Late	-15	Late
Tescl	tescalcin-like	(-3)	Early	-18	Lasting
Slitrk6	SLIT and NTRK-like family, member 6	(-8)	Early	-16	Early
Dsel	dermatan sulfate epimerase-like	(-5)	Transient	-21	Lasting
Ccdc167	coiled-coil domain containing 167	(-4)	Transient	-21	Lasting
Slc40a1	solute carrier family 40 (iron-regulated transporter), member 1	(-4)	Transient	-17	Early
Rhcg[#]	Rhesus blood group-associated C glycoprotein	(-5)	Transient	-15	Early
Tnfaip8	tumor necrosis factor, alpha-induced protein 8	(-8)	Early	-14	Early
Rsl1	regulator of sex limited protein 1	(-7)	Early	-14	Early
Fzd10	frizzled homolog 10 (Drosophila)	(-8)	Transient	-14	Early
Bola1	bolA-like 1 (E. coli)	(-4)	Transient	-14	Early
Kcne2	potassium voltage-gated channel, Isk-related subfamily, gene 2	(-4)	Early	-14	Early
Tnfrsf25	tumor necrosis factor receptor superfamily, member 25	(-4)	Early	-12	Early
Caps2	calcyphosphine 2	(-3)	Transient	-12	Early
Hes5	hairy and enhancer of split 5 (Drosophila)	(-7)	Transient	-18	Transient
Rxrg	retinoid X receptor gamma	(-7)	Transient	-14	Transient
Gria2	glutamate receptor, ionotropic, AMPA2 (alpha 2)	(-8)	Transient	-12	Transient
Ntng1	netrin G1	(-5)	Transient	-13	Transient
Gucy1a2	guanylate cyclase 1, soluble, alpha 2	(-5)	Transient	-13	Transient
Pclo	piccolo (presynaptic cytomatrix protein)	(-6)	Transient	-12	Transient
Spink8	serine peptidase inhibitor, Kazal type 8	(-4)	Transient	-13	Late

Repressions specific to P10		P10	
Gene Symbol	Gene Name	Index	Kinetic
Mkks	McKusick-Kaufman syndrome	-17	Early
Frmpd4	FERM and PDZ domain containing 4	-17	Early
Tmem107	transmembrane protein 107	-16	Early
Myh10	myosin, heavy polypeptide 10, non-muscle	-16	Early
Cttnbp2	cortactin binding protein 2	-16	Early
Zfp950	zinc finger prtoein 950	-15	Early
Zfp961	zinc finger protein 961	-15	Early
Zfp273	zinc finger protein 273	-14	Early
Zfp945	zinc finger protein 945	-13	Early
Tmem212	transmembrane protein 212	-13	Early
Gpr34	G protein-coupled receptor 34	-13	Early
Krcc1	lysine-rich coiled-coil 1	-12	Early
Pcsk2	proprotein convertase subtilisin/kexin type 2	-18	Late
Sphkap	SPHK1 interactor, AKAP domain containing	-16	Late
Kbtbd7	kelch repeat and BTB (POZ) domain containing 7	-13	Late
Tmsb15l	thymosin beta 15b like	-13	Late

Footnotes Supplementary Table 3

Index amplitudes varied from -25 to -1.5 in the whole 466 repressions recorded at P5, and from -21 to -1.4 in the 499 repressions recorded at P10. Bold characters indicate genes exhibiting R-index in the 1rst decile at the 2 ages.

Supplementary Table 4: Protein arrays at 6 and 24 h after HI in P5 and P10 brains. Values indicate fold change vs naïve animal levels. Negative values indicate decreased levels. Bold characters indicate coherent observation with transcriptome observation. Italics indicate protein variation opposite to transcriptome observations at P5 (*) and/or P10 (#).

Common to P5 and P10		P5		P10	
Gene	Protein	6h	24h	6h	24h
**Spp1**	Osteopontin (OPN)	1,59	8,33		18,99
Mpo	Myeloperoxidase		3,38		8,79
**Ccl12**	CCL12 MCP5		2,65		7,39
Igfbp1	IGFBP-1		2,35		2,04
Igfbp3	IGFBP3		2,33		2,31
Icam1	ICAM-I/CD54		2,29		2,78
**Lcn2**	Lipocalin-2/NGAL		2,00	2,12	3,73
Ccl1	CCL1-Eotaxin		1,80		6,11
Crp	C-Reactive protein	3,50	lost		1,95
C1q	C1qR1/CD93		1,96	-1,58	1,76
Il10	IL10		1,60	1,51	
Cfd	Complement factor D		1,57		1,59
Cxcl2	CXCL2/MIP-2		1,53		1,67
Il1b#	IL-1β/IL-1F2	-3,36		-1,55	
Eng	Endoglin/CD105	-2,08		-2,62	
Infl2	IL28A/B	-1,56		-1,53	
*Ccl17**	CCL17/TARC	-1,60		-1,77	
**Ccl2**	CCL2/JE/MCP1	-1,86		-1,53	
**Cd14**	CD14		2,14	-2,01	
Ccl22	CCL22/MDC		1,86	-1,79	
Ccl19	CCL19/MIP-3β		1,81	-1,72	
Tnfrsf11b	Osteoprotegerin		1,77	-1,64	
Tymp	PD-ECGF/thymidine phosphorylase		1,67	-1,82	
Cxcl1	Growth-regulated alpha protein		1,57	-1,62	
*Ldlr**#	LDL-R		1,51	-1,85	
Il6	IL6	-2,28		1,86	
**Cxcl16***	CXCL16	-2,18			1,53
Serpine1	Serpin E1/PAI-1	-1,78			2,21

Specific to P5		6h	24h
Reg3g	Reg3G	1,69	2,87
Apcs	Pentraxin 2/SAP		2,31
Il7	IL7		1,93
**Csf1**	M-CSF		1,90
Csf2	GM-CSF		1,89
Lif	LIF		1,85
Dkk1	DKK-1		1,77
Gdf15	GDF15	-3,47	-1,51
Il17a	IL17A	-8,35	
Postn	Periostin/OSF-2	-4,03	
C5	Complement C5/C5a	-2,82	
Cd160	CD160	-1,92	
Vegfa	VEGF	-1,88	
Mmp9	MMP9	-1,85	
Thpo	Thrombopoietin	-1,75	

Specifc to P10		6h	24h
**Ccl5**	CCL5/RANTES	2,17	2,96
Sele	E-selectin/CD26E	1,58	2,94
Chi3l1	Chitinase-3-like 1		4,35
Ccl6	CL6/C10		3,68
Cxcl10	CXCL10/IP10		2,32
**Angpt2**	Angiopoietin-2		2,17
Ptx3	Pentraxin 3/Tsg-14		1,92
Rarres2	Chemerin		1,90
Wisp1	WISP-1/CCN4		1,78
Lep	Leptin		1,78
Areg	Amphiregulin	-2,04	-2,05
Mmp3	MMP-3	-1,82	
